# Chromatin remodeller SMARCA4 recruits topoisomerase 1 and suppresses transcription-associated genomic instability

**DOI:** 10.1038/ncomms10549

**Published:** 2016-02-04

**Authors:** Afzal Husain, Nasim A. Begum, Takako Taniguchi, Hisaaki Taniguchi, Maki Kobayashi, Tasuku Honjo

**Affiliations:** 1 Department of Immunology and Genomic Medicine, Graduate School of Medicine, Kyoto University, Yoshida-Konoe cho, Sakyo-ku, Kyoto 606-8501, Japan; 2 Division of Disease Proteomics, Institute for Enzyme Research, University of Tokushima, Tokushima 770-8503, Japan

## Abstract

Topoisomerase 1, an enzyme that relieves superhelical tension, is implicated in transcription-associated mutagenesis and genome instability-associated with neurodegenerative diseases as well as activation-induced cytidine deaminase. From proteomic analysis of TOP1-associated proteins, we identify SMARCA4, an ATP-dependent chromatin remodeller; FACT, a histone chaperone; and H3K4me3, a transcriptionally active chromatin marker. Here we show that SMARCA4 knockdown in a B-cell line decreases TOP1 recruitment to chromatin, and leads to increases in *Igh*/*c-Myc* chromosomal translocations, variable and switch region mutations and negative superhelicity, all of which are also observed in response to TOP1 knockdown. In contrast, FACT knockdown inhibits association of TOP1 with H3K4me3, and severely reduces DNA cleavage and *Igh*/*c-Myc* translocations, without significant effect on TOP1 recruitment to chromatin. We thus propose that SMARCA4 is involved in the TOP1 recruitment to general chromatin, whereas FACT is required for TOP1 binding to H3K4me3 at non-B DNA containing chromatin for the site-specific cleavage.

## Abstract (web summary)

Topoisomerase 1 (TOP1) relieves superhelical tension when DNA strands are unwound during transcription. Here, Husain *et al*. report that SMARCA4, an ATP-dependent chromatin remodeller, is associated with TOP1 and suppresses transcription-associated genomic instability.

## Graphical abstract

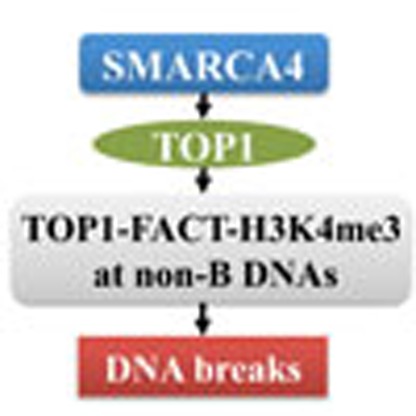

Topoisomerase 1 (TOP1) is an enzyme that relieves the superhelical tension generated when the transcription machinery travels along DNA. Normally, TOP1 nicks DNA, forms a transient covalent bond between its tyrosine residue and the 3′ phosphate of the nicked DNA, rotates DNA around the helix, and then re-ligates the cleaved ends to release negative or positive supercoils that accumulate behind or in front, respectively, of elongating RNA polymerase II (Pol II)^1^,^2^. High levels of transcription can lead to the accumulation of negative supercoils behind RNA pol II and facilitate the formation of non-B DNA structures, particularly at repeat-containing sequences. These changes in DNA structure are likely due to insufficient levels of TOP1. In fact, TOP1 reduction by small interfering RNA (siRNA) induces high levels of genomic instability^2^,^3^,^4^. The accumulated non-B DNA structures may promote genomic instability by posing steric hindrance in the rotation or re-ligation of cleaved ends following DNA nicking by TOP1, resulting in irreversible DNA cleavage^2^,^3^,^4^,^5^,^6^,^7^,^8^,^9^,^10^.

TOP1 was recently shown to be responsible for 2–5-bp deletions that occur during transcription-associated mutagenesis, and the triplet-repeat instability associated with triplet diseases such as Huntington's disease^11^,^12^,^13^,^14^. In addition, defects in DNA damage response factors involved in the processing of TOP1-DNA lesions, such as ataxia telangiectasia mutated, TDP1 (tyrosyl-DNA phosphodiesterase 1), Aprataxin and PNKP (polynucleotide kinase phosphatase), are implicated in neurodegenerative genome instability syndromes such as ataxia telangiectasia, spinocerebellar ataxia with axonal neuropathy 1, ataxia with oculomotor apraxia type 1, and microcephaly with early-onset, intractable seizures and developmental delay, respectively^15^,^16^,^17^,^18^. It is important to stress that these genome instabilities in the nerve cells do not depend on replication, but depend on transcription in which TOP1 plays a critical role.

Another enzyme associated with genomic stress is activation-induced cytidine deaminase (AID), which is normally responsible for the DNA cleavage in the switch (S) and variable (V) regions of the immunoglobulin (Ig) locus, which initiates class switch recombination (CSR) and somatic hypermutation (SHM), respectively^19^,^20^,^21^. Aberrant expression of AID induces a high frequency of mutations and chromosomal translocations in B as well as non-B cells that eventually leads to increased tumour formation^22^. It has also been shown that the activation of AID reduces TOP1 protein expression^23^, while the artificial reduction of TOP1 by siRNA augments AID-induced DNA cleavage, SHM and CSR^23^,^24^. Furthermore, the reduced expression of TOP1 in *Top1* heterozygous knockout mice leads to a dramatic increase in the frequency of SHM in Peyer's patch B cells^24^. These findings indicate that reduction in the TOP1 protein level enhances the DNA cleavage that augments SHM or CSR. However, trapping the TOP1-DNA intermediates by camptothecin inhibits both CSR and SHM, suggesting that the appropriate processing of TOP1-DNA lesions is also required for SHM and CSR. Taken together, these findings suggest that AID-dependent chromosomal translocations may serve as a model to study TOP1-mediated genomic instability.

Preferred targets of TOP1-mediated genomic instability are highly transcribed and enriched in repetitive sequences^11^,^12^,^13^. Notably, the targeted loci of AID-induced DNA cleavage also share similar properties^25^,^26^,^27^,^28^. *Ig* gene targeting by AID has been proposed to depend not only on the *cis* marking by secondary DNA structures such as non-B DNAs, produced by excessive transcription at repeat-containing AID targets, but also on *trans* marking by a variety of chromatin factors, including histone H3 tri-methyl at Lys4 (H3K4me3)^23^,^26^,^29^. Consistent with this proposal, transcription elongation factors, such as FACT (a heterodimeric complex composed of SSRP1 and SPT16/SUPT16H), SPT6/SUPT6H, SPT5/SUPT5H and H3K4me3-methyltransferases, which are required for the maintenance of H3K4me3, are essential for AID-induced genetic alterations^29^,^30^,^31^,^32^. Furthermore, non-canonical AID targets such as *MYC*, *MALAT1* and *SNHG3*, identified in a genome-wide study, are highly transcribed, flanked by repetitive sequences, and enriched with H3K4me3 as well as FACT^25^,^26^,^32^,^33^.

There is evidence that a large number of chromatin-modifying proteins are also involved in the maintenance of genomic stability. Among these, proteins found in the SWI/SNF chromatin remodelling complexes, which are also referred to as BAF (BRG1-associated factor) complexes, are frequently mutated in human cancers, suggesting that the inactivation of these proteins may induce the genomic instability that contributes to tumorigenesis^34^,^35^. However, the mechanisms by which BAF complex inactivation cause genomic instability are not well understood. The BAF complexes are highly polymorphic assemblies containing ATPase SMARCA4 (also called BRG1), which utilizes ATP hydrolysis to modulate non-covalent chromatin structural changes by destabilizing, removing, sliding and restructuring nucleosomes^36^,^37^.

To understand the molecular mechanisms involved in TOP1-mediated genomic instability, we sought to identify proteins responsible for TOP1 recruitment to chromatin. We performed TOP1 co-immunoprecipitations (co-IPs) followed by protein identification by mass spectrometry (MS) to identify proteins that associate with TOP1. Among the co-precipitated proteins, we found that SMARCA4 is involved in the recruitment of TOP1 to chromatin. Some of the recruited TOP1 interacts with the FACT that accumulates at non-B DNA containing chromatin, and forms a complex with the chromatin mark H3K4me3. Subsequently, the TOP1 complex induces breaks at non-B DNA formed at the repeat containing DNA sequences. Our findings indicate that SMARCA4 and FACT are involved in the regulation of TOP1-dependent genomic instability associated with transcription.

## Results

### Proteomic analysis of TOP1-associated proteins

We carried out co-immunoprecipitation experiments using a transfectant of the TOP1-deficient mouse B-lymphocytic leukemia cell-line P388/CPT45, that stably expresses GFP-tagged human TOP1 (GFP-TOP1) and the estrogen-binding domain (ER)-AID (AIDER) fusion protein^24^. Immunoprecipitations (IPs) were performed using the GFP-Trap system, followed by protein identification with one-dimensional SDS–PAGE (SDS polyacrylamide gel electrophoresis) separation and mass spectrometry.

**Figure 1:**
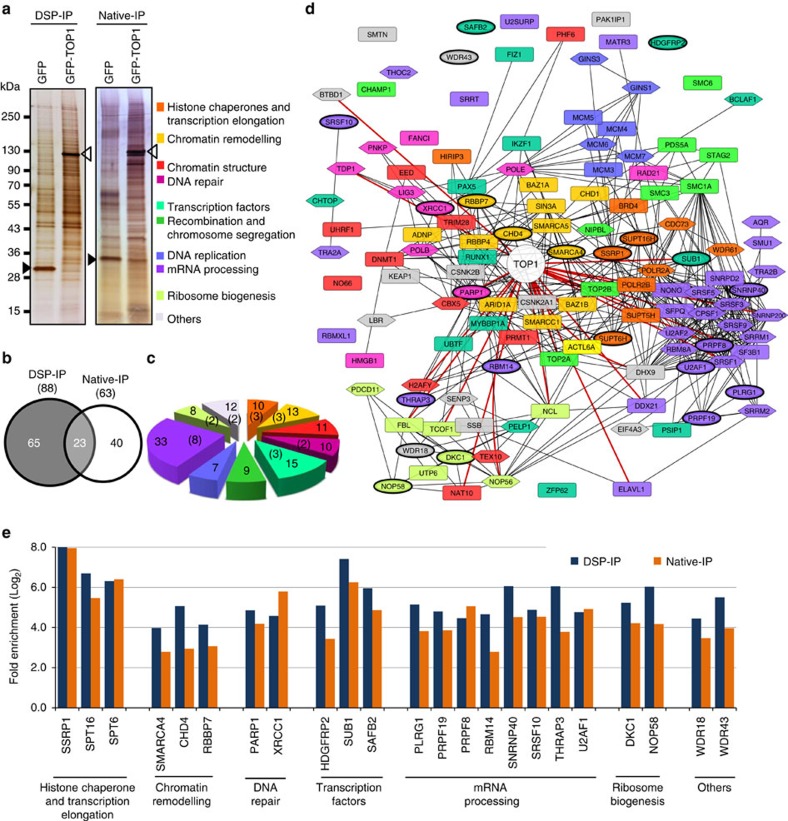
Proteome-wide identification of TOP1-associated proteins. (**a**) Representative silver-stained gels of protein fractions obtained from GFP-TOP1 IPs performed under native (Native-IP) or DSP-cross-linked conditions (DSP-IP). Immunoprecipitations were performed with GFP-Trap_A using nuclear extracts prepared from 2 × 10^8^ DSP treated or untreated P388/CPT45 cells expressing either GFP (P388/CPT45-GFP) or GFP-TOP1 (P388/CPT45-GFP-TOP1) as described in the ‘Methods'. The IPed proteins were electrophoresed on SDS–PAGE gel (4–20% acrylamide gel) and visualized with SilverQuest Silver Staining Kit (Invitrogen). Open and closed triangles represent the protein bands corresponding to GFP-TOP1 or GFP alone, respectively. For MS analysis, IPed proteins were processed as described in the ‘Methods'. (**b**) *Venn* diagram representing LC-MS/MS identified TOP1-associated proteins obtained by DSP- or native-IP. The number of proteins identified by each method is shown in parentheses. The complete list of MS identified proteins is shown in Supplementary Tables 1 and 2. (**c**) Functional classification of TOP1-associated proteins. The number of common proteins identified by both methods in the various groups is shown in parentheses. (**d**) Physical and functional interactions among the TOP1-associated proteins based on information obtained from the STRING 9.1 database ( http://string-db.org). Proteins identified by both methods are shown in ellipses, whereas those identified by either DSP-IP or native-IP are shown in rectangles or hexagons, respectively. Red coloured edges represent known TOP1 associations identified in the STRING database. Proteins that are not connected with rest of the network showed no evidence of association with TOP1 or the other network proteins in the STRING database. The colour of each node represents the functional class shown on the left. (**e**) Fold enrichment of the common proteins identified by GFP-TOP1 IP compared with their levels in GFP IPs from control cells. The fold enrichments of all the proteins identified by each method are shown in Supplementary Tables 1 and 2. DSP, dithiobis-succinimidyl propionate.

We used dithiobis-succinimidyl propionate (DSP), a cell-permeable and thiol-reversible chemical crosslinker, to identify labile but functionally relevant TOP1-interacting proteins. To generate a real-time snapshot of the TOP1-protein interactome *in vivo*, AID-activated P388/CPT45-GFP-TOP1 transfectants were treated with DSP (0.6 mM) for 30 min, followed by IP (DSP-IP) and MS-identification of the immunoprecipitated (IPed) proteins (Fig. 1a–d). Under these conditions, TOP1 was efficiently crosslinked to its interacting partners, and the IPed fraction contained only the crosslinked form of TOP1, which was thiol-reversible (Supplementary Fig. 1a,b). To overcome the shortcomings of DSP-IP, such as nonspecific protein trapping, we also performed IP without crosslinking (native-IP), although proteins with labile TOP1 interactions may be lost with this method (Fig. 1a–d; Supplementary Fig. 1c). We postulated that molecules commonly trapped by both methods may be a more reliable representation of the TOP1 interactome than molecules identified by only one method.

The MS analysis of proteins obtained by DSP- and native-IP from GFP-TOP1-expressing cells led to the identifications of 88 and 63 proteins, respectively (Supplementary Tables 1 and 2). Among the co-IPed proteins, 65 proteins were identified by DSP-IP alone, 40 proteins were identified by native-IP alone, and 23 proteins were identified by both methods (Fig. 1b). Theoretically, all the proteins that were identified by native-IP should be picked up by DSP-IP due to their further stabilization by crosslinking. However, despite stabilization of the labile interactions, crosslinking negatively affects the extraction of many protein complexes likely due to their crosslinking with other non-soluble proteins such as those in the nuclear matrix, and thus limits their successful IP and identification by MS. The functional classification of the 128 unique TOP1-associated proteins suggests that TOP1 may be a central component of a large protein interaction network associated with transcription, DNA repair and other chromatin-related functions (Fig. 1c).

To further elucidate the functional relationships among the TOP1-associated proteins, we mapped them using the STRING database, a knowledge base of protein associations, and visualized the interactions by Cytoscape 3.2 (refs 38, 39). As shown in Fig. 1d, 113 (88%) of these proteins were connected by a functional network, suggesting that the TOP1-associated proteins were functionally interrelated. Notably, only 28 of the 113 proteins in this network were previously known to associate with TOP1; these included SRSF1, NCL, ELAVL1, FBL, NONO, SFPQ, TCOF1, PARP1 and BTBD1. One of the major pathways implicated in the repair of DNA damage induced by TOP1-DNA adducts requires proteolysis of TOP1 by proteasome, excision by TDP1, followed by repair that requires the action of XRCC1 complex proteins such as PNKP, PARP1, LIGIII and POLB. Interestingly, all of these were identified in our proteomic studies (Supplementary Tables 1 and 2)^40^. These results indicate that the experimental strategies described here identified both known and novel TOP1-associated proteins.

### TOP1-associated proteins are required for efficient CSR

**Figure 2:**
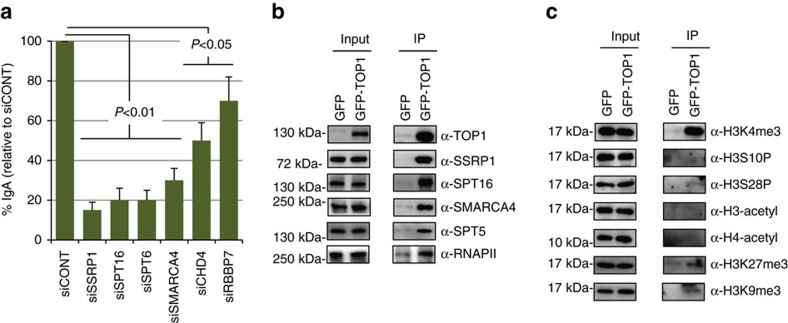
TOP1-associated proteins are required for efficient CSR. (**a**) Effect of siRNA-mediated KD of histone chaperones and chromatin remodellers on IgM to IgA switching in CH12F3-2A cells. After electroporation of the indicated siRNAs, cells were cultured for 24 h, and then stimulated by CIT for another 24 h before FACS analysis. Data are presented as % IgA switching relative to the IgA switching in cells transfected with control siRNA (siCONT) with similar GC content. The data represents the mean of the three independent experiments with standard deviations. Statistical significance as evaluated by Student's *t*-test is shown. (**b**) Confirmation of association of TOP1 with FACT, SMARCA4 and other transcription-associated proteins. The nuclei from 2 × 10^8^ P388/CPT45 transfectants expressing either GFP (P388/CPT45-GFP) or GFP-TOP1 (P388/CPT45-GFP-TOP1) were IPed with GFP-Trap_A as described for native-IP in the ‘Methods', and 5–10% of the IPed proteins were electrophoresed on 4–20% SDS–PAGE gradient gel, followed by western blot with indicated antibodies. (**c**) Association of TOP1 with H3K4me3 and other histone PTMs. Immunoprecipitated proteins obtained in **b** were immunoblotted to test for the presence of the indicated histone-PTMs using their specific antibodies. The position of molecular weight markers is shown on the left of each western blot image.

We focused on the proteins identified by both methods, which included histone chaperones and transcription elongation factors (SSRP1, SPT16, SPT6), chromatin remodelling complex proteins (SMARCA4, CHD4 and RBBP7), DNA repair proteins (PARP1 and XRCC1) and proteins involved in splicing and ribosome biogenesis (Fig. 1e). In addition to SMARCA4, other components of the BAF complex such as SMARCC1, ARID1A and ACTL6A were also identified by DSP-IP (Supplementary Table 1). To investigate the functional relevance of these proteins in TOP1 recruitment, we performed siRNA-mediated knockdown (KD) of these proteins in CH12F3-2A cells, and assessed the effects on IgM to IgA switching in response to stimulation with CD40L, IL4, and TGFβ (CIT) (Fig. 2a). We were particularly interested in the proteins involved in chromatin modification because of their likely involvement in recruiting TOP1 to chromatin. The KD of the transcription elongation-associated histone chaperones, SPT6 and FACT (SSRP1 and SPT16) strongly reduced CSR as previously shown^29^,^30^,^41^. The KD of the chromatin remodelling proteins SMARCA4, CHD4 and RBBP7 led to 70%, 50% and 30% reductions in CSR, respectively (Fig. 2a; Supplementary Fig. 2a–c). Thus, we concentrated on the role of SMARCA4 and FACT in TOP1 recruitment, as SPT6 is known to directly regulate AID expression.

We confirmed that both SMARCA4 and FACT (SSRP1 and SPT16 subunits) were specifically IPed from GFP-TOP1-expressing cells, but not from control cells expressing GFP alone (Fig. 2b; Supplementary Fig. 1d–f). In addition, we confirmed the interaction of TOP1 with other transcription-associated proteins, such as RNA pol II and SPT5, a subunit of the DSIF (DRB sensitivity inducing factor) complex, which were also found in the proteomic analysis, albeit with lower enrichment. These results not only validated the proteomic data, but also suggested that recruitment of TOP1 may be transcription-dependent. Consistent with this notion, treating CH12F3-2A cells with the transcription inhibitors DRB (5,6-Dichloro-1-β-D-ribofuranosylbenzimidazole, 25 μM for 24 h) or ActD (actinomycin D, 2 μM for 4 h) dramatically decreased the TOP1-ChIP (chromatin immunoprecipitation) signal at the *Igh* locus (Supplementary Fig. 3). To gain insight into the role of epigenetic marking on TOP1 recruitment, the TOP1-co-IPed proteins were analysed for the presence of several histone post-translational modifications (PTMs). We found that TOP1-associated chromatin was highly enriched in H3K4me3 as compared with other tested histone-PTMs (H3-acetyl, H4-acetyl, H3S10P, H3S28P, H3K27me3 and H3K9me3; Fig. 2c). These data suggest that TOP1 may preferentially bind to H3K4me3, and be likely enriched in the H3K4me3-modified genomic regions.

### SMARCA4 depletion augments AID-induced genomic instability

The KD of SMARCA4 in CH12F3-2A cells with three individual siRNAs or their pool inhibited IgA switching without causing significant cell death (Supplementary Figs 2a–c and 4a–d), and reduced the expression of α-germline transcripts (GLTs) (Supplementary Fig. 2d). Drastic reduction of SMARCA4 by siRNA had a very limited effect on cell proliferation, suggesting that CSR inhibition upon SMARCA4 KD is largely due to the inhibition of αGLT expression (Supplementary Fig. 2d,e). To further confirm this possibility, we performed the CSR assay using an artificial switch substrate in AIDER expressing NIH3T3 cells^42^. SMARCA4 KD in these cells reduced neither CSR nor the expression of Sμ or Sα transcripts (Pre-Tr1 and Pre-Tr2) from the artificial promoters (Supplementary Fig. 2f–h). The data further confirmed that CSR defect upon SMARCA4 KD in CH12F32-A cells is due to the inhibition of αGLT expression.

**Figure 3:**
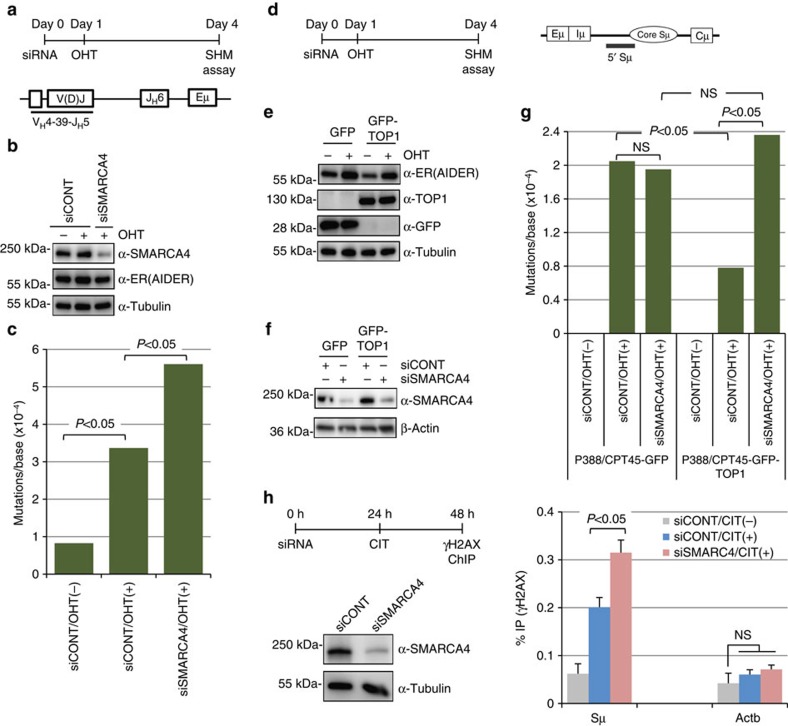
SMARCA4 depletion augments SHM in a TOP1-dependent manner. (**a**) Top: experimental design for SHM analysis of the V region in BL2-AID^−/−^AIDER cells. Bottom: schematic representation of recombined V(D)J region of BL2-AID^−/−^AIDER cells. The region analysed for SHM is underlined. (**b**) Confirmation of siRNA-mediated KD of SMARCA4 in BL2-AID^−/−^AIDER cells. Expression of AIDER is also shown. Tubulin is used as loading control. (**c**) Mutation frequency in the V region after 3 days of AIDER activation by OHT treatment. Details of the mutation analysis are shown in Supplementary Tables 3 and 4. (**d**) Left: schematic illustration of the experimental design for SHM analysis of the Sμ region in P388/CPT45 cells. Right: schematic representation of Sμ region of P388/CPT45 cells. The 5′ Sμ region analysed for SHM is underlined. (**e**) Expression of GFP or GFP-TOP1, and AIDER in P388/CPT45 cells expressing either GFP (P388/CPT45-GFP) or GFP-TOP1 (P388/CPT45-GFP-TOP1) is analysed by western blotting with indicated antibodies. (**f**) Confirmation of siRNA-mediated KD of SMARCA4 in P388/CPT45 cells by western blotting with indicated antibodies. Expression of β-actin is shown as loading control. (**g**) Mutation frequency in the Sμ region after 3 days of AIDER activation by OHT. Details of the mutation analysis are shown in Supplementary Tables 5 and 6. (**h**) Left: Schematic representation of the γH2AX-ChIP assay using CH12F3-2A cells (top), and confirmation of SMARCA4 KD by western blot with anti-SMARCA4 antibody. Expression of tubulin is shown as a loading control. Right: γH2AX-ChIP signal in Sμ region is represented as the fraction of immunoprecipitated DNA (%IP) normalized to the total amount of DNA used for immunoprecipitation. γH2AX-ChIP signal at β-actin (Actb), an AID non-targeted gene, is used as a negative control. The data represents the mean of the three independent experiments with standard deviations. Statistical significance as evaluated by Fisher's exact test (**c**,**g**) or Student's *t*-test (**h**) is shown. The position of molecular weight markers is shown on the left of each western blot image. AIDER, AID fused with the hormone-binding domain of the estrogen receptor (ER); NS, not significant; OHT, 4-hydroxytamoxifen; SHM, somatic hypermutation.

We next analysed the role of SMARCA4 in SHM. To determine the effect of SMARCA4 KD on V region SHM, we used AID-knockout BL2 cells (human Burkitt's lymphoma line) expressing AIDER (BL2-AID^−/−^AIDER)^43^,^44^. After transfection with siRNA-targeting human SMARCA4, the cells were treated with 4-hydroxytamoxifen (OHT) from days 1 to 4 to activate AID, and the 426-bp rearranged V_H_4–39-J_H_5 region was sequenced (Fig. 3a). Transfection of SMARCA4 siRNA sufficiently suppressed SMARCA4 protein expression, as compared with the effect of control non-targeting siRNA with a similar GC content (Fig. 3b). The KD of SMARCA4 increased the SHM frequency in the V(D)J region induced by AIDER activation (Fig. 3c; Supplementary Tables 3 and 4).

As the enhancement of SHM by SMARCA4 KD is reminiscent of that induced by TOP1 depletion, we speculated that SMARCA4 depletion in BL2 cells may affect TOP1 function, which led to enhanced DNA cleavage and SHM. To test this possibility, we analysed the effect of SMARCA4 KD on SHM in the Sμ region of the TOP1-deficient P388/CPT45 cells expressing either GFP (P388/CPT45-GFP) or GFP-TOP1 (P388/CPT45-GFP-TOP1; Fig. 3d–g; Supplementary Tables 5 and 6). Introducing SMARCA4 siRNA into these cells efficiently reduced the SMARCA4 protein expression (Fig. 3f). As reported previously^24^, expression of GFP-TOP1 in the TOP1-deficient P388/CPT45 cells resulted in a clear reduction in SHM frequency (Fig. 3g). Notably, SMARCA4 depletion rescued the decrease in SHM frequency in the P388/CPT45-GFP-TOP1 cells, suggesting that SMARCA4 reduction counteracts TOP1 overexpression. Interestingly, SHM augmentation by SMARCA4 KD was not observed in TOP1-deficient P388/CPT45-GFP cells, confirming that SMARCA4 depletion and TOP1 deficiency are functionally related. Since increased mutations may represent increments in the double-stranded breaks (DSBs), we analysed the effect of SMARCA4 depletion on the H2AX phosphorylation. Indeed, depletion of SMARCA4 in CH12F3-2A cells resulted in the increased formation of AID-induced γH2AX foci in the Sμ region (Fig. 3h).

**Figure 4:**
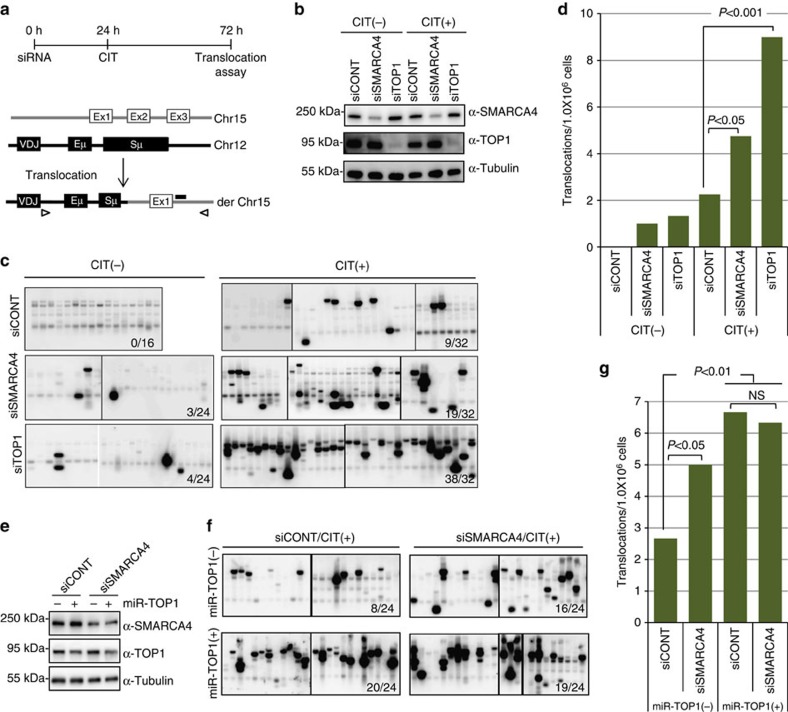
SMARCA4 depletion augments chromosomal translocations in a TOP1-dependent manner. (**a**) Top: schematic illustration of experimental design for translocation assay in CH12F3-2A cells. Bottom: PCR amplification scheme to detect *Igh*/*c-Myc* chromosomal translocations. Triangles represent the positions of primers used to amplify the rearranged regions. The position of the *Myc* probe used in the Southern blot hybridization is shown as a horizontal black bar. (**b**) Confirmation of SMARCA4 or TOP1 KD by their respective siRNAs in CH12F3-2A cells used in the translocation assay shown in **c** and **d**. Expression of tubulin is shown as loading control. (**c**) Southern blot analysis of PCR-amplified fragments with *Myc*-specific probe. The CH12F3-2A cells were transfected with the indicated siRNAs and cultured with (+) or without (−) CIT stimulation. (**d**) Frequency of *Igh*/*c-Myc* chromosomal translocations derived from the indicated samples. (**e**) Confirmation of SMARCA4 or TOP1 KD in CH12F3-2A cells expressing Tet-inducible microRNA targeting *Top1* mRNA (miR-TOP1). SMARCA4 or TOP1 were KD either by the transfection of the CH12F3-2A cells by SMARCA4 siRNA or by the treatment with tetracycline (50 nM), respectively. The (+) or (−) signs indicate the presence or absence of Tet-inducible miR-TOP1. Expression of tubulin is shown as loading control. Genomic DNAs isolated from these cells were used for the translocation assays shown in **f** and **g**. (**f**) Southern blot analysis of PCR-amplified fragments with *Myc*-specific probe. (**g**) Frequency of *Igh*/*c-Myc* chromosomal translocations derived from the SMARCA4- and/or TOP1-depleted CH12F3-2A cells. The numbers at bottom-right corners of the blots indicate the number of the translocations detected in total number of PCR reactions. Statistical significance as evaluated by Fisher's exact tests for indicated sets of data is shown. The position of molecular weight markers is shown on the left of each western blot image. CIT, CD40L-IL4-TGFβ; NS, not significant.

As TOP1 reduction is known to cause genomic instability, we assessed the effect of SMARCA4 KD on the chromosomal translocations involving the *c-Myc* oncogene and *Igh* locus, a hallmark of various B-cell malignancies. The products of long-range PCR amplification of the translocated genomic DNA using *Igh*- and *c-Myc*-specific primers were detected by Southern blotting with *Myc* probes (Fig. 4a). Depletion of either SMARCA4 or TOP1 with siRNA in CH12F3-2A cells led to dramatically increased translocation frequencies (Fig. 4b–d); however, the frequency of translocations upon TOP1 KD was higher than that observed upon SMARCA4 KD. This conclusion is further confirmed by the KD of SMARCA4 with the pool of three SMARCA4 siRNAs (Supplementary Fig. 4f). In addition, although SMARCA4 KD augmented the chromosomal translocation frequency in TOP1-expressing cells, it had no effect on chromosomal translocation in CH12F3-2A cells expressing microRNA directed against *Top1* mRNA (Fig. 4e–g). To exclude the possibility that absence of the chromosomal translocation increase by SMARCA4 KD in TOP1-depleted cells is not due to saturation of the assay sensitivity, we performed the translocation assay using lesser amount of genomic DNA (300 ng instead of 750 ng per PCR reaction). Even under this unsaturated assay conditions, we failed to see additive increments in the *Igh/c-Myc* translocations (Supplementary Fig. 5). These results confirmed that the effect of SMARCA4 KD on chromosomal translocations was dependent on the presence of TOP1. In addition, we also showed that complementation of siRNA-mediated KD of SMARCA4 by the co-transfection of plasmid expressing siRNA-resistant WT-SMARCA4 abolished the increments in *Igh/c-Myc* translocations (Supplementary Fig. 6).

### FACT is essential for AID-induced genomic instability

**Figure 5:**
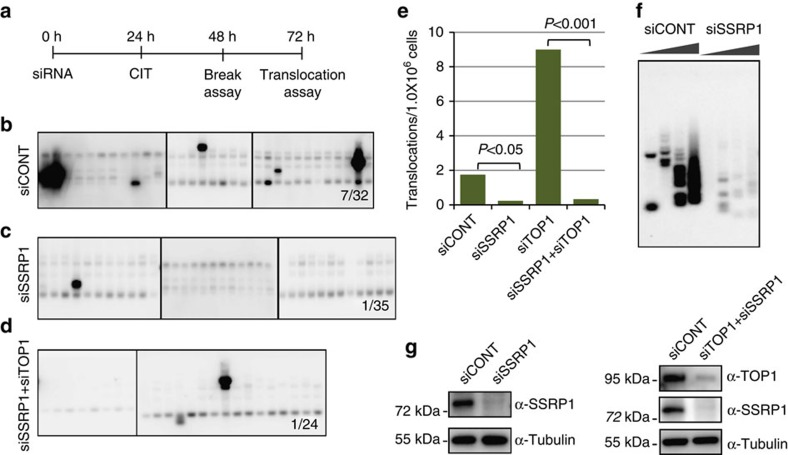
FACT is essential for AID-induced genomic instability even in the TOP1-deficient cells. (**a**) Schematic illustration of experimental design for the analysis of DNA double-strand breaks by ligation mediated PCR (LM-PCR) and *Igh/c-Myc* chromosomal translocations in CH12F3-2A cells. (**b**–**d**) Southern blot analysis of PCR-amplified fragments with *Myc*-specific probe using genomic DNA prepared from CH12F3-2A cells transfected with the indicated siRNAs. The numbers at bottom-right corners of the blots indicate the number of the translocations detected in total number of PCR reactions. (**e**) Frequency of *Igh*/*c-Myc* chromosomal translocations derived from the indicated samples. The value of translocation frequency upon TOP1-KD is taken from Fig. 4c. (**f**) LM-PCR analysis of the AID-induced DNA double-strand breaks in the Sμ region of CH12F3-2A cells following transfection with the indicated siRNAs. Wedges indicate a threefold increase in DNA amount. (**g**) Confirmation of single or simultaneous KD of SSRP1 and TOP1 in CH12F3-2A cells. Expression of tubulin is shown as loading control. Genomic DNAs isolated from these cells were used for the translocation assays and LM-PCR analysis. Statistical significance as evaluated by Fisher's exact test is shown. CIT, CD40L-IL4-TGFβ.

We next investigated the role of FACT, another prominent component of the TOP1 complex, and found that FACT depletion in CH12F3-2A cells caused a dramatic reduction in the *Igh*/*c-Myc* chromosomal translocations induced by AID expression (Fig. 5a–c,e,g). The results suggest that unlike TOP1 and SMARCA4, FACT facilitates AID-induced genomic instability. To further confirm this possibility, we assayed the AID-induced *Igh*/*c-Myc* chromosomal translocations upon simultaneous KD of TOP1 and FACT in CH12F3-2A cells. Surprisingly, FACT KD abolished the TOP1 depletion-mediated augmentation of *Igh*/*c-Myc* chromosomal translocations (compare Fig. 4c versus Fig. 5d,e). When we measured formation of DSBs using ligation-mediated PCR, FACT depletion severely reduced the formation of AID-induced DSBs, which is consistent with our previous studies^29^ (Fig. 5f). These data suggest that FACT expression is essential for DNA cleavage at *Ig* and *Myc* loci, even in TOP1-deficient cells. Thus, FACT and SMARCA4 play distinct roles in AID-induced genomic instability.

### SMARCA4 depletion reduces TOP1 recruitment to chromatin

**Figure 6:**
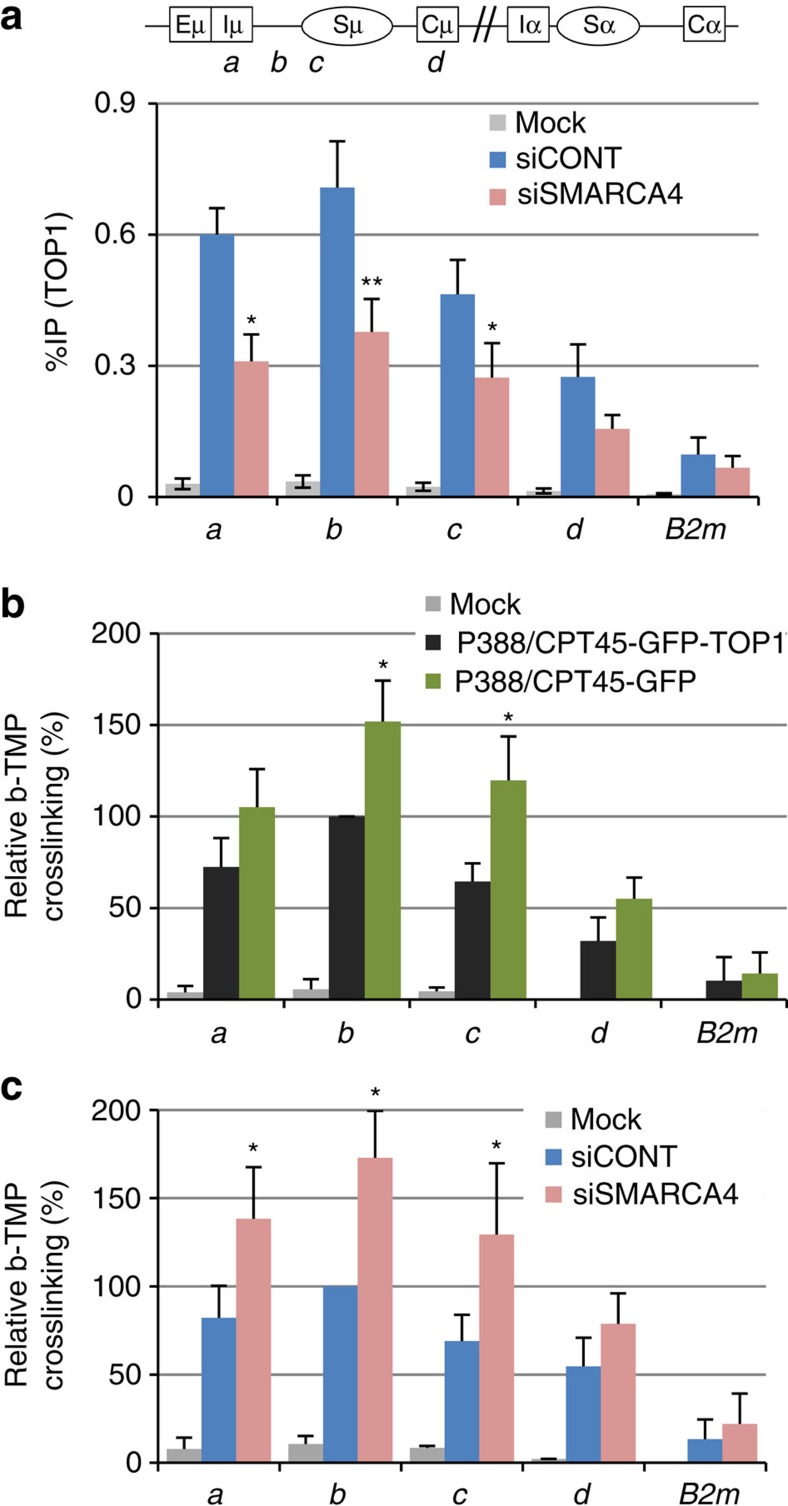
SMARCA4 is required for efficient TOP1 recruitment to chromatin. (**a**) Schematic diagram showing the position of the ChIP assay PCR products in *Igh* locus. Bottom view shows the TOP1-ChIP assay following SMARCA4 KD in CH12F3-2A cells. Before lysis, the cells were pretreated with 50 nM of bortezomib for 3 h. ChIP data are normalized as in Fig. 3h, and represents the mean of the four independent experiments with standard deviations. (**b**,**c**) Analysis of negative superhelicity in P388/CPT45 cells expressing GFP or GFP-TOP1 (**b**), and CH12F3-2A cells after SMARCA4 KD (**c**). ChIP data is shown as the fraction of immunoprecipitated DNA (%IP) normalized to the input DNA signals, and the maximum value in each data set was set as 100%. The data represent the mean of the three independent experiments with standard deviations. The mock data show background values from control IP with no antibody. Asterisks (* and **) denote statistically significant differences with *P≤*0.05 and *P≤*0.01, respectively, as determined by Student's *t*-test. *B2m*, beta-2-microglobulin; b-TMP, biotin-trimethylpsoralen.

Since TOP1 is required for augmentation of SHM and chromosomal translocations by SMARCA4 depletion, we speculated that SMARCA4 is involved in TOP1 deposition at the *Igh* locus, and thus we performed TOP1-ChIP using CH12F3-2A cells following transfection with SMARCA4 siRNA. Indeed, SMARCA4 depletion reduced deposition of TOP1 in the Sμ region, confirming that TOP1 association with SMARCA4 is functionally relevant and required for efficient TOP1 deposition at the *Igh* locus (Fig. 6a). The reduction in the recruitment of TOP1 upon SMARCA4 KD was further confirmed by TOP1-ChIP analysis upon SMARCA4 KD by the pool of three SMARCA4 siRNAs (Supplementary Fig. 4e). The reduction in association of TOP1 with chromatin causes the accumulation of negative supercoils behind elongating RNA Pol II, resulting in the formation of non-B DNA structures as well as single-stranded patches of DNA^4^,^9^,^10^,^23^,^45^. To evaluate the relative frequencies of negative superhelicity, we used biotin-trimethylpsoralen (bTMP), which preferentially intercalates into under-wound DNA, followed by ultraviolet-crosslinking, streptavidin-based IP of the crosslinked DNA, and quantification of the IPed DNA by quantitative PCR (qPCR; Supplementary Fig. 7a). The ultraviolet-dependent incorporation of bTMP into genomic DNA was confirmed by a dot blot (Supplementary Fig. 7b). Using this method, we found that more bTMP was incorporated into the Sμ region of TOP1-deficient P388 cells (P388/CPT45-GFP), compared with cells expressing exogenous TOP1 (P388/CPT45-GFP-TOP1), indicating that S-region DNA is more negatively supercoiled in TOP1-deficient cells (Fig. 6b). Similarly, SMARCA4 KD in CH12F3-2A cells also led to the enhanced accumulation of negative supercoils (Fig. 6c). These results further confirm the involvement of SMARCA4 in recruitment of TOP1, which suppresses the formation of non-B DNA structures by correcting uneven distribution of superhelix.

**Figure 7:**
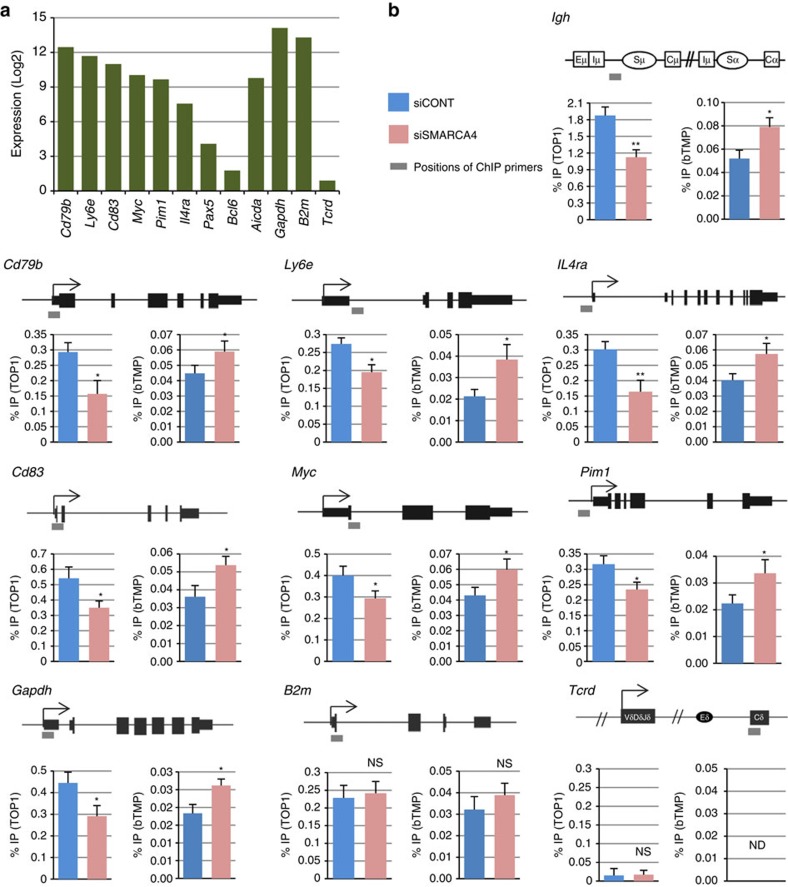
SMARCA4 is required for TOP1 recruitment and maintenance of negative superhelicity at non*-Igh* targets of AID. (**a**) Expressions of non*-Igh* AID targets, *Aicda*, and AID non-targeted genes derived from microarray analysis of CIT stimulated CH12F3-2A cells. (**b**) Top: schematic diagram showing the position of the ChIP assay PCR products (underlined bar) in the respective *Igh*, non*-Igh* and AID non-targeted genes. Bottom: analysis of TOP1-ChIP (left) and negative superhelicity (right) following SMARCA4 KD in CH12F3-2AA cells. For TOP1-ChIP assay, before lysis, the cells were pretreated with 10 μM of camptothecin for 30 min. ChIP data are normalized as in Fig. 3h, and represent the mean of the three independent experiments with standard deviations. Asterisks (* and **) denote statistically significant differences with *P≤*0.05 and *P≤*0.01, respectively, as determined by Student's *t*-test. *B2m*, beta-2-microglobulin; *Gapdh*, glyceraldehyde-3-phosphate dehydrogenase; ND, not detected; NS, not significant; *Tcrd*, T-cell receptor delta chain.

Next, we analyse the effect of SMARCA4 depletion on TOP1 recruitment and negative supercoiling at non*-Igh* AID target loci identified by whole-genome sequencing^46^,^47^,^48^. Among these; *Pim1, Cd83, Cd79b, Ly6e, IL4ra* and *Myc* are efficiently expressed in CH12-F3-2A cells (Fig. 7a). As shown in Fig. 7b, the depletion of SMARCA4 by siRNAs resulted in the decrements in the TOP1 recruitment to these loci. Consistently, we found that SMARCA4 depletion leads to the increment in the incorporation of bTMP, a marker for an increment in negative supercoils (Fig. 7b). Interestingly, neither TOP1 recruitment nor bTMP incorporation was observed at a non-transcribed gene (*Tcrd)*, indicating a requirement of transcription for both TOP1 recruitment and accumulation of negative supercoils. Together, these data show that SMARCA4 is a general recruiter of TOP1, and required to maintain B- DNA structures.

As depletion of SMARCA4 caused reduction in TOP1 recruitment to non-*Igh* AID target loci, we analysed the formation DSBs at these loci using γH2AX-ChIP assays. The depletion of SMARCA4 resulted in the increased DSBs at these loci (Supplementary Fig. 8a,b). Next, we investigated the H2AX phosphorylation by flow cytometry to assay the effect of SMARCA4 depletion on the global DSB formation. As shown in Supplementary Fig. 8c,d, we could not see large changes in the γH2AX level upon SMARCA4 depletion both in the absence or presence of AID, suggesting that TOP1 and transcription-dependent genomic damage is less frequent than those induced by DNA damage-inducing drugs. However, it is noteworthy that SMARCA4 KD almost doubled the percentage of cells with high level of γH2AX (Supplementary Fig. 8d).

### ATPase activity of SMARCA4 is essential for TOP1 recruitment

**Figure 8:**
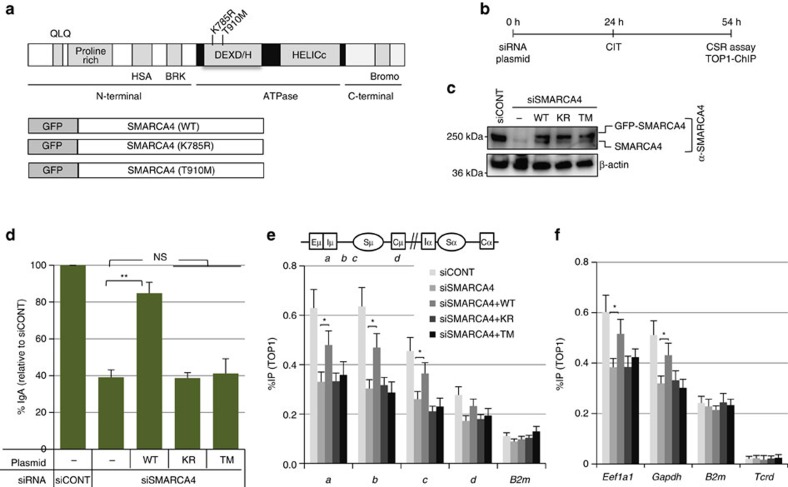
ATPase activity of SMARCA4 is essential for efficient TOP1 recruitment. (**a**) Top: schematic representation of the human SMARCA4 protein showing the positions of the mutations in the analysed mutants. Bottom: schematic representation of GFP-fused WT- or mutated-SMARCA4 proteins. (**b**) Schematic representation of the experimental design for the CSR complementation and TOP1-ChIP assays. (**c**) Confirmation of the siRNA-mediated KD of endogenous mouse SMARCA4, and expression of GFP-tagged human SMARCA4 (WT, and KR and TM mutants). Expression of β-actin is shown as loading control. (**d**) CSR complementation assay using CH12F3-2A cells expressing siRNA-resistant human SMARCA4. Data are presented as % IgA switching relative to the IgA switching in cells transfected with control siRNA (siCONT) with similar GC content. The data represent the mean of the three independent experiments with standard deviations. (**e**,**f**) TOP1-ChIP assays following CSR complementation in CH12F3-2A cells. Before lysis, the cells were pretreated with either 50 nM of bortezomib for 3 h (**e**) or 10 μM of camptothecin for 30 min (**f**). Top view shows schematic diagram with the position of the ChIP assay PCR products in *Igh* locus. ChIP data were normalized as in Fig. 3h, and represent the mean of the three independent experiments with standard deviations. Asterisks (* and **) denote statistically significant differences with *P≤*0.05 and *P≤*0.01, respectively, as determined by Student's *t*-test. The position of molecular weight markers is shown on the left of each western blot image. *B2m*, beta-2-microglobulin; CIT, CD40L-IL4-TGFβ; CSR, class switch recombination; *Eef1a1*, eukaryotic translation elongation factor 1 alpha 1; *Gapdh*, glyceraldehyde-3-phosphate dehydrogenase; NS, not significant; *Tcrd*, T-cell receptor delta chain.

To confirm the requirement of ATPase activity of SMARCA4 in TOP1 recruitment, we analysed the effect of two SMARCA4 point mutants: K785R (KR) and T910M (TM), both of which are incorporated normally into the BAF complex, but whose ATPase activity is highly compromised (Fig. 8a)^49^. First, we performed the CSR complementation assay by introducing siRNA-resistant GFP-tagged human wild-type (WT) or mutated SMARCA4 into CH12F3-2A cells, along with SMARCA4 siRNAs to deplete endogenous SMARCA4 (Fig. 8b). SMARCA4-targeted siRNA treatment decreased the expression of the endogenous SMARCA4, but not of the exogenous GFP-tagged SMARCA4 (Fig. 8c). CSR was rescued in cells expressing WT SMARCA4, but not in cells expressing the KR or TM mutant (Fig. 8b–d). Similarly, using TOP1-ChIP, we found that the defective TOP1 loading at the *Igh* locus in SMARCA4-depleted cells was significantly restored by expressing the siRNA-resistant WT SMARCA4, but not the KR or TM mutant (Fig. 8e). These results showed that the ATPase activity SMARCA4 is required for efficient CSR as well as for the recruitment of TOP1 to the *Igh* locus.

To know whether ATPase activity of SMARCA4 is also required for TOP1 recruitment to other loci, we performed TOP1-ChIP upon SMARCA4 depletion in CH12F3-2A cells pretreated by camptothecin (CPT), and found that SMARCA4 KD reduces the amount of TOP1 bound to chromatin at variety of tested loci even in the presence of CPT (Figs 7b and 8f). In addition, we also found that WT but not ATPase dead SMARCA4 proteins could rescue the defect in TOP1 recruitment in the presence of CPT (Fig. 8f). Taken together, these data suggest that SMRACA4 ATPase activity is required for TOP1 trapping both in the absence (Fig. 8e) and presence of CPT (Figs 7b and 8f).

### FACT is required for the association of TOP1 with H3K4me3

**Figure 9:**
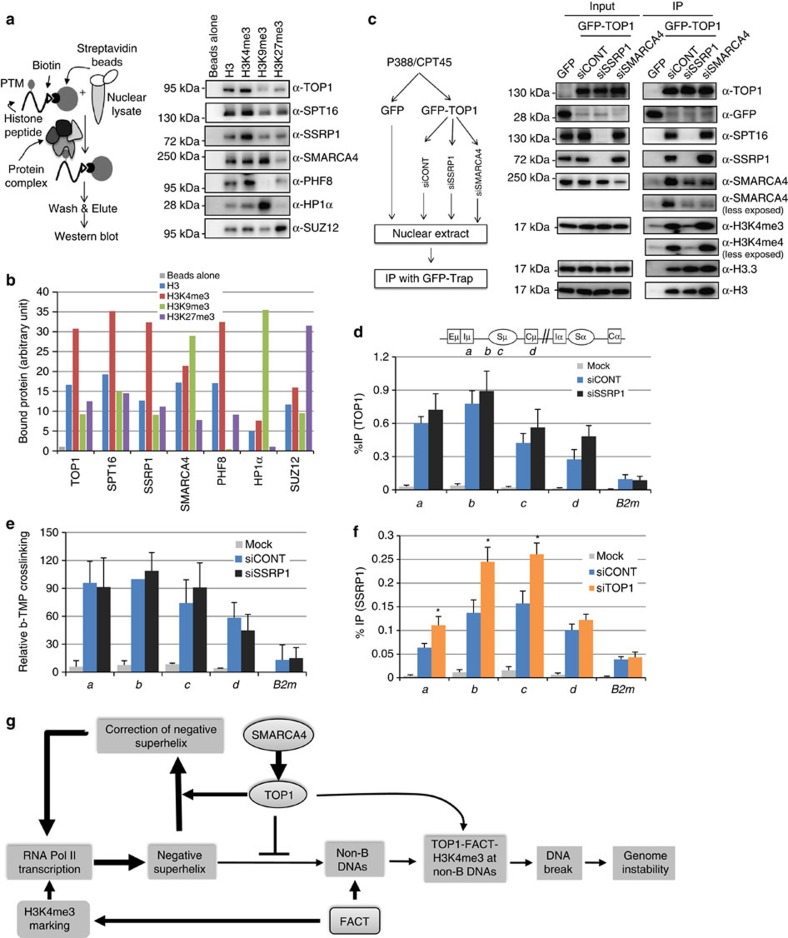
FACT is required for TOP1-H3K4me3 complex formation. (**a**) Histone peptide pull-down assays using biotinylated peptides carrying either H3K4me3 or other histone PTMs. Cell lysates for peptide pull-down assays from CIT-stimulated CH12F3-2A cells were prepared as described for native-IP (see ‘Methods'). Pulled-down proteins were immunoblotted with the indicated antibodies. (**b**) Densitometric quantification of western blot images shown in Fig. 9a is performed to analyse relative binding of the indicated proteins with the histone peptides. (**c**) TOP1 co-IP following SSRP1 or SMARCA4 KD in P388/CPT45-GFP-TOP1 cells. Cell lysates prepared form P388/CPT45-GFP-TOP1 cells transfected with the indicated siRNAs were subjected to immunoprecipitation with GFP-Trap_A, and the IPed proteins were western blotted with the indicated antibodies. (**d**) TOP1-ChIP analysis following SSRP1 KD in CH12F3-2A cells. Before lysis, the cells were pretreated with 50 nM of bortezomib for 3 h. ChIP data were normalized as in Fig. 3h. The data represent the mean of the three independent experiments with standard deviations. The mock data show background values from control IP with no antibody. (**e**) Analysis of negative superhelicity in the SSRP1-depleted cells. ChIP data were normalized as in Fig. 6c. (**f**) Analysis of SSRP1-ChIP following TOP1 KD in CH12F3-2A cells. ChIP data are normalized as in Fig. 3h, and represent the mean of the three independent experiments with standard deviations. Asterisks (*) denote statistically significant differences with *P≤*0.05, as determined by Student's *t*-test. The position of molecular weight markers is shown on the left of each western blot image. *B2m:* beta-2-microglobulin; b-TMP, biotin-trimethylpsoralen. (**g**) Diagrammatic representation of role of SMARCA4, FACT and H3K4me3 in TOP1-mediated genomic instability. High levels of transcription lead to the accumulation of negative superhelix. SMARCA4 recruits TOP1 to actively transcribed chromatin to correct excessive accumulation of negative superhelix and prevent the formation of non-B DNAs at repetitive sequences. Reduction in TOP1 recruitment causes accumulation of negative superhelix leading to the formation of non-B DNA structures at repetitive sequences. On the other hand, FACT binds to non-B DNA through SSRP1, and traps a fraction of SMARCA4-recruited TOP1, which facilitates the formation of a complex of TOP1, FACT and H3K4me3 at non-B DNA containing chromatin, resulting in the DNA cleavage.

To examine the role of another TOP1-binding protein, H3K4me3, in TOP1 recruitment, we performed peptide pull-down assays by incubating nuclear extracts of CH12F3-2A cells, which were prepared under native-IP conditions, with biotinylated histone H3 tail peptides carrying H3K4me3 or other histone PTMs. Peptide-bound proteins were collected by streptavidin beads and subjected to western blotting (Fig. 9a). The results showed the preferential binding of TOP1 to H3K4me3 peptides, as compared with unmodified H3, H3K9me3 or H3K27me3 peptides (Fig. 9a,b). Notably, each of the FACT subunits (SSRP1 and SPT16) showed relatively strong binding to H3K4me3 compared with the other peptides, indicating that TOP1, FACT and H3K4me3 are likely to form a complex (Fig. 9a,b). In contrast, SMARCA4 showed stronger binding to H3K9me3 than H3K4me3. To confirm the biological activity of the biotinylated histone peptides, the bound fractions were also tested for the presence of known histone-PTM readers, and shown to contain PHF8, HP1α and SUZ12 in the complexes bound to H3K4me3, H3K9me3 or H3K27me3, respectively^50^.

To further ascertain whether FACT is involved in binding of TOP1 to H3K4me3, we performed TOP1-IP from P388/CPT45-GFP-TOP1 cells following siRNA-mediated depletion of SSRP1 or SMARCA4, and evaluated the association of TOP1 with H3K4me3 (Fig. 9c). As previously reported^51^, both SSRP1 and SPT16 proteins were significantly depleted by the transfection of siRNA targeting SSRP1 alone (Fig. 9c). Notably, TOP1-IP from FACT-depleted cells failed to pull down H3K4me3, confirming that FACT is indeed essential for the binding of TOP1 to H3K4me3 (Fig. 9c). However, despite the absence of FACT, interaction of TOP1 with histone H3 and H3.3 remained intact, suggesting that FACT-independent TOP1 associations with other forms of chromatin also exist. In contrast to the effects of FACT KD, SMARCA4 KD did not affect TOP1 binding to H3K4me3 or FACT. However, in the absence of FACT, the amount of SMARCA4 associated with TOP1 was decreased, suggesting that FACT may be involved in stabilizing the TOP1–SMARCA4 complex. We then analysed the ability of TOP1 to directly bind H3K4me3 using purified recombinant human TOP1 protein and customized histone tail peptide arrays. We found that TOP1 did not bind H3K4me3 directly, but showed a weak affinity for N-terminal acetylated histone H3 and H4 peptides, confirming that binding of TOP1 to H3K4me3 was indirect (Supplementary Fig. 9).

Next, we performed TOP1-ChIPs to assess the effect of FACT KD on the recruitment TOP1 to the *Igh* locus in CH12F3-2A cells. In contrast to the drastic effect of SMARCA4 KD, FACT KD did not significantly affect TOP1 recruitment to the *Igh* locus (Fig. 9d). These results suggest that only a small fraction of the total TOP1 is associated with FACT and H3K4me3 at the *Igh* locus, although the majority of TOP1 is recruited by SMARCA4. Indeed, the interaction of TOP1 with Histone H3 and H3.3 remained intact even in the absence of FACT (Fig. 9c). Consistent with these findings, FACT KD did not cause any significant change in the accumulation of negative superhelicity (Fig. 9e). To the contrary, FACT appears to accumulate at non-B DNA containing chromatin as indicated by the increased chromatin binding of FACT upon TOP1 depletion (Fig. 9f). As FACT KD caused severe defects in AID-induced DNA breaks, it is likely that FACT recruits TOP1 to form the TOP1–FACT complex at non-B DNA containing chromatin, resulting in the irreversible DNA cleavage at non-B DNA (Fig. 9g). In fact, SSRP1 was originally identified as a protein recognizing a specific structure of DNA^52^,^53^.

## Discussion

Mutations or changes in the expression of SMARCA4 or other BAF complex subunits are associated with a wide variety of cancers, including lymphoid malignancies. Recent cancer genome sequencing studies showed that ∼20% of human cancers harbour mutations in at least one subunit of the BAF complex, suggesting a role of the SMARCA4-containing BAF complexes in the suppression of genomic instability^34^,^35^. However, the mechanisms linking the inactivation of BAF complex subunits with the induction of genomic instability are not well understood.

A recent study suggested that SMARCA4 is required for Topoisomerase-2α-mediated decatenation of the newly replicated sister chromatids during cell division^49^. On the other hand, the present study revealed the role of SMARCA4 in the efficient recruitment of TOP1 during transcription, and showed that SMARCA4 depletion mimics TOP1 deficiency in AID-induced genomic instability. Since TOP1 deficiency as well as defects in the processing of TOP1-DNA lesions are widely implicated in transcription-associated genomic instability and neurodegenerative diseases^3^,^4^,^5^,^16^,^18^, it is possible that the genomic instability induced by SMARCA4 inactivation in human cancers is due, at least in part, to the compromised TOP1 recruitment to chromatin.

Recurrent chromosomal translocations involving *Ig* loci and proto-oncogenes such as *c-MYC*, *BCL-2*, *BCL-6* and *FGFR* are hallmarks of B-cell malignancies, and are associated with Burkitt's lymphoma, follicular lymphoma, diffuse large cell lymphoma and multiple myeloma, respectively^54^,^55^,^56^. We observed a dramatic increase in the frequency of chromosomal translocations involving *Igh* and *c-Myc* loci in SMARCA4 or TOP1-deficient cells, indicating that defects in the efficient recruitment of TOP1 in SMARCA4-depleted cells induce genomic instability. Notably, even in the absence of AID activation, a low frequency of chromosomal translocations was observed in SMARCA4- or TOP1-depleted CH12F3-2A cells. In addition, SMARCA4 depletion also reduces the TOP1 occupancy and enhances negative supercoiling at non-*Igh* AID target loci. However, it remains to be studied whether depletion of SMARCA4 or TOP1 enhances mutations or chromosomal translocations involving these loci.

Although FACT and H3K4me3 also formed a complex with TOP1, their functions were distinct from that of SMARCA4. We found that in the absence of FACT, TOP1 failed to associate with H3K4me3, indicating that TOP1 association with the H3K4me3-associated protein complex was FACT-dependent (Fig. 9c). In addition, we found that FACT depletion almost completely abolished the generation of AID-induced S region breaks as well as *Igh*/*c-Myc* translocations. The strong inhibition of AID-induced breaks by FACT KD was probably due to the defect of TOP1 association with the H3K4me3–FACT complex, which may be involved in determining cleavage sites. Although FACT depletion disturbed the association of TOP1 with H3K4me3, it did not affect the binding of TOP1 with chromatin through H3 or H3.3. Thus, it is likely that FACT binds to only a small fraction of SMARCA4-recruited TOP1 to form complex with H3K4me3 probably at non-B DNA. Indeed, the depletion of TOP1, which induces the formation of non-B DNAs, resulted in the increased chromatin binding of FACT, indicating that FACT has an affinity for non-B DNAs (Fig. 9f). In fact, SSRP1, a subunit of FACT, was originally identified as a protein recognizing a specific DNA structure^52^,^53^. In contrast, SMARCA4 depletion suppressed the recruitment of TOP1 to chromatin, but had little effect on the association TOP1 with FACT and H3K4me3. Thus, our findings showed that SMARCA4 is involved in the general recruitment of TOP1 to chromatin, while a limited amount of TOP1 recruited by SMARCA4 is sufficient to promote DNA cleavage through the interaction with FACT accumulated at the non-B DNA (Fig. 9f,g; Supplementary Table 7).

High transcriptional levels are associated with mutations, deletions, recombination and other deleterious genetic events that can threaten genomic stability^5^,^57^. TOP1 and transcription-induced non-B DNA structures formed at repetitive or palindromic sequences are involved in transcription-associated mutagenesis, unstable triplet repeat diseases and AID-induced *Ig* gene diversification^11^,^12^,^13^,^23^,^24^. In addition, there is evidence that FACT and H3K4me3 are involved in AID-induced genetic alterations^25^,^29^,^30^. Here, we showed that KD of TOP1 or SMARCA4 increased the accumulation of negative supercoils, and promoted V and S region DNA cleavage, resulting in increased frequencies of SHM and *Igh*/*c-Myc* chromosomal translocations. Notably, the simultaneous depletion of SMARCA4 and TOP1 did not show additive effects on SHM or *Igh*/*c-Myc* translocations, suggesting that the functions of these two factors may overlap. Taken together, these results suggest that the SMARCA4 KD-mediated augmentation of SHM or *Igh*/*c-Myc* translocations was due to inefficient TOP1 recruitment to the S and *Myc* regions. In addition, we showed that the ATPase activity of SMARCA4, which is essential for chromatin remodelling, was required for efficient TOP1 recruitment.

The accumulation of unwound DNA in SMARCA4- or TOP1-depleted cells increased the frequency of *Igh*/*c-Myc* translocations, even in the absence of AID induction, suggesting that the excessive accumulation of non-B DNA promotes DNA cleavage. On the other hand, the depletion of FACT, which was required for H3K4me3 marking and for the association of TOP1 with H3K4me3 did not affect the accumulation of non-B DNA, but completely abolished the AID-induced DNA cleavage and *Igh*/*c-Myc* translocations. Notably, TOP1 KD, which normally results in the accumulation of non-B DNA structures, failed to induce *Igh*/*c-Myc* translocations in FACT-depleted cells (Fig. 5a–g). These data suggest that the accumulation of non-B DNA structures promotes AID-induced DNA cleavage only in the presence of FACT. Taken together, these observations suggest that AID-induced genetic alterations and transcription-dependent genomic instability share a common mechanism involving the accumulation of non-B DNA and the recruitment of TOP1 to chromatin, which is mediated by SMARCA4, FACT and H3K4me3.

In conclusion, we showed that TOP1 is recruited by SMARCA4 to chromatin so that transcription-associated uneven distribution of superhelix is corrected. A small fraction of TOP1 binds to FACT that is enriched at non-B DNA containing chromatin, and forms a complex with H3K4me3, which is essential for AID-induced DNA cleavage (Fig. 9g). Depletion of FACT dissociates TOP1 from H3K4me3-associated complex, and abolishes AID-induced DNA cleavage. Deficiency of either TOP1 or SMARCA4 reduces TOP1 binding to chromatin, and promotes the formation of non-B DNA structures, but a small amount of TOP1 can still bind to FACT and H3K4me3 at non-B DNA containing chromatin, and promotes genomic instability.

## Methods

### Cell lines and RNAi oligonucleotide transfection

The human Burkitt's lymphoma line BL2, the mouse B-cell lymphoma line CH12F3-2A expressing *Bcl2*, and the TOP1-deficient P388/CPT45 cells expressing GFP (P388/CPT45-GFP) or GFP-tagged human TOP1 (P388/CPT45-GFP-TOP1) were previously described^24^,^29^,^44^. The BL2 and P388/CPT45 cells also express AIDER, a fusion protein in which AID is fused to the hormone-binding domain of the estrogen receptor (ER), which is activated by treating cells with OHT. Chemically modified Stealth siRNA oligonucleotides (Invitrogen) were introduced into cells using the Nucleofector 96-well electroporation system (Lonza) to knockdown the expression of specific genes. After electroporation, the cells were cultured for 24 h, and then stimulated by CIT (CD40L, IL4 and TGFβ) or OHT (1 μM), and cultured for another 24–72 h before collection and analysis. The list of antibodies, primers and Stealth siRNAs used in this study is shown in Supplementary Tables 8–10.

### Analysis of CSR and SHM

CH12F3-2A cells were CIT-stimulated for 24–48 h to induce CSR, and surface expression of IgM and IgA was analysed by staining with FITC-conjugated anti-mouse IgM (eBioscience) and PE-conjugated anti-mouse IgA (Southern Biotechnology Associates) antibodies, respectively. Flow cytometry analysis were performed using a Becton-Dickinson FACS-Calibur, as previously described^30^. Analysis of CSR in NIH 3T3 cells expressing artificial switch substrate SCI (μ, α) and AIDER was performed as described earlier^42^. Briefly, NIH 3T3 cells were transfected with control siRNA or human SMARCA4 siRNA using Lipofectamine 2000 (Invitrogen). Twenty-four hours post transfection, cells were stimulated with 1 μM of OHT for 48 h, and stained with allophyco-cyanin-conjugated anti-CD8α (eBioscience).

To analyse the effect of SMARCA4 depletion on SHM in BL2 cells expressing AIDER, the cells were transfected with SMARCA4 siRNA, cultured for 24 h and then treated with OHT (1 μM) for 72 h, followed by total RNA extraction using TRIzol (Invitrogen). The cDNA was synthesized from 0.5–1 μg of total RNA using Oligo d(T)-primers and SuperScript III (Invitrogen). PCR fragments corresponding to the 426-bp rearranged V_H_4–39-J_H_5 region were cloned into the pGEM-T Easy Vector (Promega). The 408-bp sequence between the two primers was analysed for the presence of mutations^58^. Analysis of somatic mutations in the S-region of the P388/CPT45 cells was performed as previously described^24^. The mutation frequency was calculated as the number of mutations per total bases from independent experiments. The primers used for SHM analysis are shown in Supplementary Table 8.

### Co-immunoprecipitation

TOP1-deficient mouse B-cell lymphoma cell line P388/CPT45 expressing AIDER, and either GFP or GFP-TOP1 was used to co-IP TOP1-associated proteins. Chemical crosslinking-based IP (DSP-IP) was performed using DSP (Thermo Scientific)^59^. Cells (2 × 10^8^) were incubated in phosphate-buffered saline (PBS) containing 0.6 mM DSP for 30 min at 4 °C with mild rotation. After removing PBS, the remaining DSP was quenched by incubating the cells in TBS (50 mM Tris-Cl pH 7.4, 150 mM NaCl, 1 mM EDTA) for 15 min on ice. To prepare nuclear extracts, cells were subjected to hypotonic lysis, and then the isolated nuclei were lysed in lysis buffer (50 mM Tris-Cl pH 7.5, 150 mM NaCl, 0.25% Triton X-100, 0.25% Na-deoxycholate, 0.05% SDS, 1 mM EDTA, 5% glycerol) containing 5 mM MgCl_2_, 500 U ml^−1^ of Benzonase nuclease (Novagen), 300 mM NaCl, and protease inhibitor cocktail (Roche). The nuclear extract was then sonicated for 10 min, followed by rotation for 1 h at 4 °C and centrifugation at 20,000*g* for 10 min. The supernatant was pre-cleared with blocked agarose beads (Chromotek) and then incubated with GFP-Trap_A (Chromotek) for 2 h at 4 °C. The agarose beads were washed sequentially with TBS, lysis buffer, high-salt lysis buffer (500 mM NaCl) and TBS. Finally, the beads were re-suspended in 100 μl 2 × SDS-sample buffer containing 5% β-mercaptoethanol, and heated for 10 min at 95 °C to elute the bound proteins.

To perform IP without crosslinking (native-IP), the nuclei prepared from 2 × 10^8^ cells were suspended in HGN165 buffer (20 mM HEPES pH 7.4, 10% glycerol, 165 mM NaCl) containing 2 mM MgCl2, 1 mM DTT, 500 U ml^−1^ of Benzonase nuclease, 0.1% Triton X-100 and protease inhibitor cocktail, followed by mild sonication to rupture the nuclei and rotation at 4 °C for 1 h. After collecting the supernatant, the insoluble pellet was sequentially extracted with the above-mentioned buffer containing a higher concentration of Triton X-100 (0.3%), followed by extraction with buffer containing a higher concentration of NaCl (200 and 250 mM). The four extracts were pooled, centrifuged and the supernatants were IPed using GFP-Trap_A. The beads were washed extensively with HGN165 buffer, and the bound fractions were eluted as described above. To perform reverse co-IP, HEK 293T cells were transfected with 10 μg of pEGFP-C2-SSRP1 or pEGFP-C2-SMARCA4 plasmids, and 48 h post transfection, the cells were collected and nuclear extracts were IPed as described for native-IP. The eluted proteins were subjected to western blotting with specific antibodies. Less-cropped western blots are shown in Supplementary Fig. 10.

### Proteomic analysis

For quantitative analysis of crosslinked IP samples, ^18^O post-labelling method was used^60^. Briefly, 130 μg samples obtained from the crosslinked IP experiments were digested with 1:50 modified porcine trypsin (Promega) in the presence of 8 M urea and 100 mM Tris-HCl (pH 8.0) after reduction with 2 mM dithiothreitol and alkylation with 10 mM iodoacetamide. The reaction was stopped with 1% TFA, and cleaned using a C18 cartridge (Bond Elute C18 EWP, Agilent). The eluted peptides were divided and dried completely in a Speed-Vac concentrator (Thermo Fisher). One half of the dried peptides were labelled with ^18^O and the rest with ^16^O by incubating with 1:50 trypsin/peptide (w/w) in 100 μl of 50 mM NH_4_HCO_3_ buffer made either in H_2_^18^O or in H_2_^16^O containing 10 mM CaCl_2_. After incubating for 5 h at 37 °C with shaking, the reaction was stopped by boiling the sample in a 100 °C water bath for 20 min, followed by immediate freezing at −80 °C. Equal amounts of TOP1 and control IP samples were mixed and fractionated over a strong cation exchange column^61^. To compensate for the incomplete labelling, TOP1 sample was labelled with ^18^O and control sample was labelled with ^16^O in one sample set, while the labelling was reversed in the second set. Twenty-two fractions obtained were separately subjected to the LC-MS/MS (liquid chromatography tandem mass spectrometry) analysis, using nano high-performance liquid chromatography (LC Packings, UltiMate 3000) interfaced online to a hybrid Fourier-transform ion cyclotron resonance mass spectrometer (Thermo Fisher, LTQ-FT). The details of the analytical conditions were as described previously^62^. The data were processed using a Mascot Distiller software (ver. 2.3.2, Matrix Science, UK), and subjected to database search (Mascot, version 2.3, Matrix Science) against *Mus musculus* protein sequences in the Swiss-Prot database. Mascot distiller was also used for the quantification of proteins.

Owing to the limited amounts of samples obtained, semi-quantitative analysis was used for native-IP experiments. Briefly, 30 μg of immunoprecipitated protein samples were electrophoresed on SDS–PAGE gels (4–20% acrylamide gel), and the gel was stained with Coomassie brilliant blue. Each lane was cut into 15 slices of equal size, destained and subjected to in-gel digestion with trypsin as described previously^63^. The obtained peptides were subjected to LC-MS/MS analyses as described above.

### Histone peptide pull-down assay

The nuclear extracts for histone peptide pull-down (PPD) assays were prepared as described for native-IP. For PPD assay, 40 μl of streptavidin Dynabeads M-280 (Invitrogen) were coupled to 20 μg of biotinylated histone peptides and then incubated with a nuclear extract equivalent to 2 × 10^7^ cells for 2 h at 4 °C. The magnetic beads were washed extensively with HGN165 buffer, and peptide-bound proteins were eluted by heating the beads in 100 μl of 2 × SDS-sample buffer containing 5% β-mercaptoethanol at 95 °C for 10 min. The eluted proteins were subjected to western blotting with specific antibodies. Information about the peptides used for PPD assay is shown in Supplementary Table 11.

### Analysis of *Igh*/*c-Myc* chromosomal translocations

The *Igh*/*c-Myc* translocation junctions (derivative chromosome 15) were PCR-amplified from genomic DNA obtained from 48 h CIT-stimulated CH12F3-2A cells using the Expand Long Template PCR System^64^. A total of 16 to 48 aliquots of genomic DNA were analysed in separate reactions. The conditions for both first and second round of PCR were as follows: 94 °C for 3 min, followed by 25 cycles at 94 °C for 15 s; 62 °C for 15 s; 68 °C for 7.5 min and a final extension of 5 min at 68 °C. The PCR products were electrophoresed on ethidium bromide-containing 1% agarose gels and subjected to Southern blotting with *Myc*-specific probe. The primer and probe sequences are shown in Supplementary Table 8.

### Analysis of DNA double-strand breaks

Genomic DNAs were prepared from CH12F3-2A cells stimulated with CIT for 24 h. DSBs were analysed by ligation-mediated PCR as previously described^65^,^66^. Briefly, living CH12F3-2A cells were isolated by Percoll density gradient centrifugation and embedded in low-melting-point agarose followed by DNA extraction within low-melting agarose plugs. A total of 20 μl of genomic DNA was ligated with linker in a final volume of 100 μl. Ligation reaction was stopped by adding 200 μl of H_2_O followed by heating at 70 °C for 10 min. Threefold dilutions of input DNA were PCR amplified by KOD-FX-Neo polymerase (TOYOBO). The amount of ligated DNA in the input was normalized with *Gapdh* DNA. PCR products were electrophoresed on 1% agarose gels followed by Southern blotting with 5′ Sμ probe. The primer and probe sequences are shown in Supplementary Table 8.

### Chromatin immunoprecipitation

The γH2AX-ChIP assay was performed using ChIP-IT Express Kit (Active Motif) as previously described^29^. For TOP1-ChIP, CIT-stimulated CH12F3-2A cells (3 × 10^6^) were treated with either 50 nM of Bortezomib (Janssen Pharmaceuticals) for 3 h or 10 μM of camptothecin (Calbiochem) for 30 min. The cells were rapidly lysed in 0.9 ml of 10 mM Tris-Cl pH6.8, 20 mM EDTA, 4% Triton X-100, 6M guanidinium isothiocyanate, 1% Sarkosyl and 60 mM DTT. The genomic DNA was precipitated by addition of 0.6 ml of 100% ethanol followed by centrifugation^67^. The genomic DNA was sheared using components ChIP-IT Express Kit (Active Motif), and 20 μg of genomic DNA was immunoprecipitated with 3 μg of TOP1 antibody and Protein G-coupled magnetic beads (Active Motif). The beads were sequentially washed by a standard series of wash buffers (low salt, high salt, LiCl and Tris/EDTA), and the IPed chromatin was eluted with 1% SDS and 0.1 M NaHCO_3_ and purified using phenol–chloroform. The locus-specific enrichment was detected by qPCR, and normalized to the input DNA. The primers and antibodies used are shown in Supplementary Tables 8 and 9, respectively.

### Analysis of negative superhelicity

To identify regions of negative superhelicity, we partially adapted a biotin-trimethylpsoralen (bTMP)-based assay described elsewhere^68^. Biotin-TMP was synthesized by the biotinylation of 4′-aminomethyltrioxsalen (Sigma). Briefly, cells were treated with 30 μg ml^−1^ bTMP for 20 min in the dark and then ultraviolet crosslinked at 360 nm for 10 min. Genomic DNA was isolated using SDS and proteinase K digestion followed by phenol–chloroform extraction. Ultraviolet-dependent bTMP crosslinking of genomic DNA was confirmed by dot blot using HRP-conjugated streptavidin as a probe. For ChIP analysis, the sheared DNA was IPed with streptavidin Dynabeads M-280 (Invitrogen). The beads were washed as described above and the IPed DNA was eluted by boiling the beads in 95% formamide containing 10 mM EDTA for 10 min at 90 °C. The eluted fractions were pooled and purified using the Qiagen PCR purification kit. The locus-specific enrichment was detected by qPCR normalized to the input DNA. A list of the primers used is shown in Supplementary Table 8.

### Analysis of cell proliferation

The effect of SMARCA4 depletion on CH12F3-2A cell proliferation was analysed using CellTrace CFSE Cell Proliferation Kit (Molecular Probes)^69^. Briefly, CH12F3-2A cells were transfected with siSMARCA4 or control siRNA (siCONT), and immediately labelled with 5 μM of CFSE (carboxyfluorescein diacetate succinimidyl ester) for 15 min at 37 °C. CIT was added 24 h later and FACS analysis was performed at 24 h post stimulation on a Becton-Dickinson FACS-Calibur flow cytometer. The cells treated with Aphidicolin (2 μg ml^−1^), a well-known inhibitor of cell cycle progression, were used as a positive control.

### Flow cytometry analysis of γH2AX phosphorylation

FACS-based H2AX phosphorylation assay kit (Millipore, catalogue # 17–344) was used to stain γH2AX in CH12F3-2A cells, essentially as described by the manufacturer. The cells were analysed on a Becton-Dickinson FACS-Calibur flow cytometer.

### Construction of plasmids

The human SMARCA4 cDNA was PCR amplified using the pCMV5-BRG1-FLAG plasmid (Addgene#17873) as a template, and cloned into the SalI/SmaI sites of the pEGFP-C2 vector. Constructs expressing mutant SMARCA4 (K785R or T910M) with defective ATPase activity were prepared by amplifying the WT construct using the indicated mutagenesis primers (Supplementary Table 8). Plasmid for the expression of GFP-SSRP1 was previously described^29^. These constructs were used for CSR rescue experiments (see below) and reverse co-IPs in HEK 293T cells.

### CSR complementation assay

To evaluate the requirement for ATPase activity of SMARCA4 in CSR, 2 μg of the siRNA-resistant-WT or -mutant SMARCA4 expression plasmids (described above) were co-transfected along with SMARCA4 siRNA oligonucleotides into CH12F3-2A cells. The cells were cultured for 24 h and then treated with CIT for 24–48 h. CSR complementation was analysed as described previously^29^.

## Additional information

**How to cite this article:** Husain, A. *et al*. Chromatin remodeller SMARCA4 recruits topoisomerase 1 and suppresses transcription-associated genomic instability. *Nat. Commun.* 7:10549 doi: 10.1038/ncomms10549 (2016).

## Supplementary Material

Supplementary Information Supplementary Figures 1-10 and Supplementary Tables 1-11
