# Supplementary material for: Chromatin remodeller SMARCA4 recruits topoisomerase 1 and suppresses transcription-associated genomic instability
Source: Nat Commun. 2016 Feb 4;7:10549. doi: 10.1038/ncomms10549 (PMC4742980; doi:10.1038/ncomms10549)
Supplement: Supplementary Information — Supplementary Figures 1-10 and Supplementary Tables 1-11 [file ncomms10549-s1.pdf]

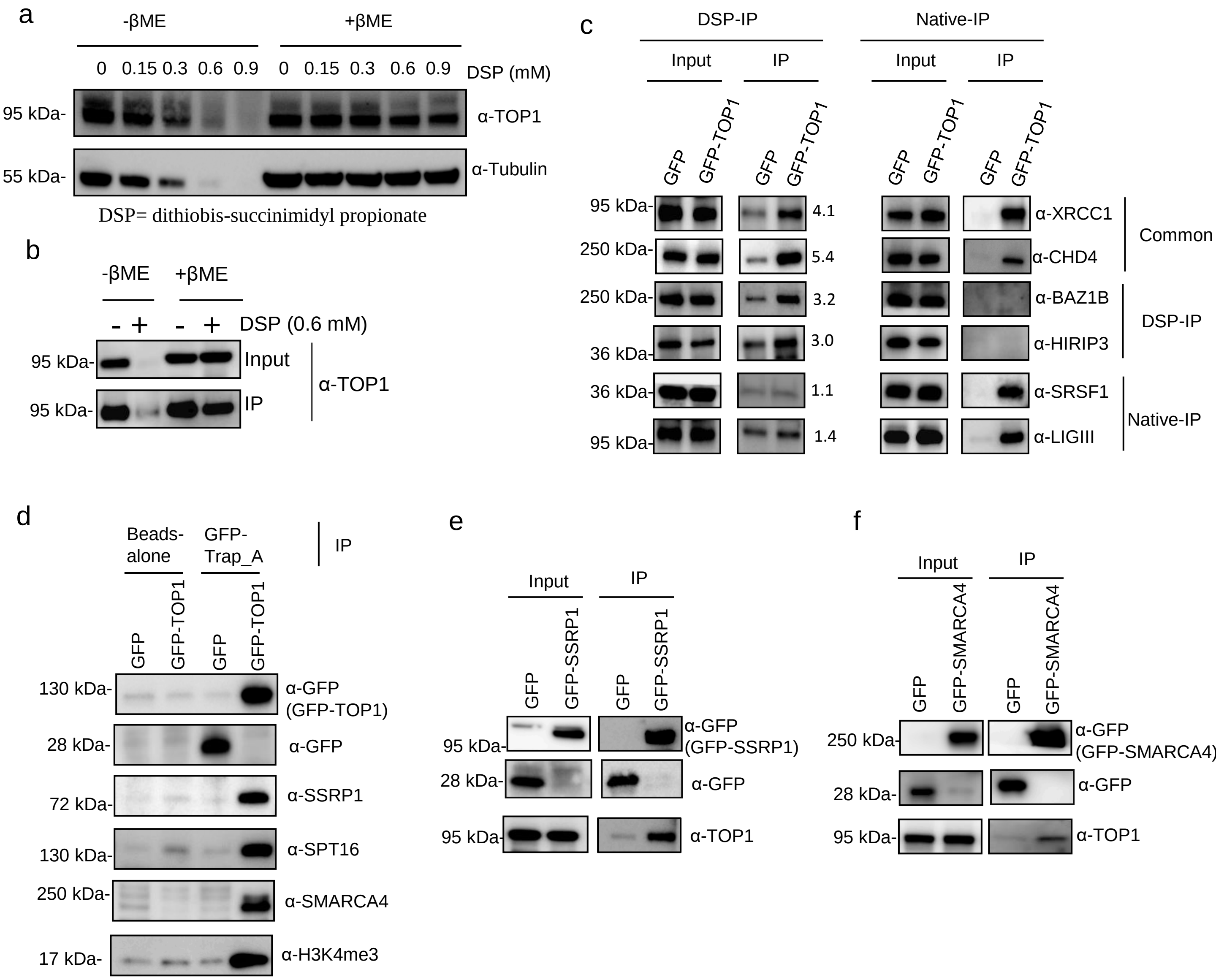

Supplementary Figure 1. Supporting information related to the identification and analysis of TOP1-associated proteins, related to Figs 1 and 2. (a) Optimization of DSP-crosslinking conditions to co-IP TOP1-associated proteins. Whole cell lysates prepared from the cells treated with the indicated concentration of DSP for 30 min were boiled in 1× SDS-sample buffer with (+) or without (-) 2.5% β-mercaptoethanol (β-ME), and subjected to Western blotting with the indicated antibodies. (b) Confirmation of co-IP of cross-linked and thiol-reversible TOP1-associated proteins. Proteins were IPed with GFP-Trap®_A from P388/CPT45-GFP-TOP1 cells, and eluted by boiling the beads in 2× SDS-sample buffer with (+) or without (-) 5% β-ME. The eluted proteins were immunoblotted with the indicated antibodies. (c) Confirmation of association of TOP1 with selected proteins identified by DSP-IP, native-IP, or both methods. The number next to DSP-IP panel shows the relative enrichment of IPed proteins as determined by densitometric quantitation. (d) Analysis of non-specific binding of SSRP1, SPT16, SMARCA4, and H3K4me3 to the agarose beads not coupled to GFP antibody. Beads alone control were purchased from Chromotek (binding control agarose beads, bab-20), and were identical to GFP-Trap®_A agarose beads except that the latter is coupled to GFP antibody. P388/CPT45-GFP-TOP1 nuclear extracts prepared as in native-IP were IPed with GFP-Trap®_A or beads alone, and IPed proteins were immunoblotted with the indicated antibodies. (e-f) Analysis of association of TOP1 with FACT or SMARCA4 by inverse co-IP using nuclear extracts prepared from HEK 293T cells transfected with plasmids expressing GFP-SSRP1 or GFP-SMARCA4. HEK 293T cells transfected with plasmid expressing GFP alone were used as control. Nuclear extracts prepared as in native-IP condition were IPed with GFP-Trap®_A agarose beads, and IPed proteins were analyzed by Western blotting with the indicated antibodies. The position of molecular weight markers is shown on the left of each western blot image.

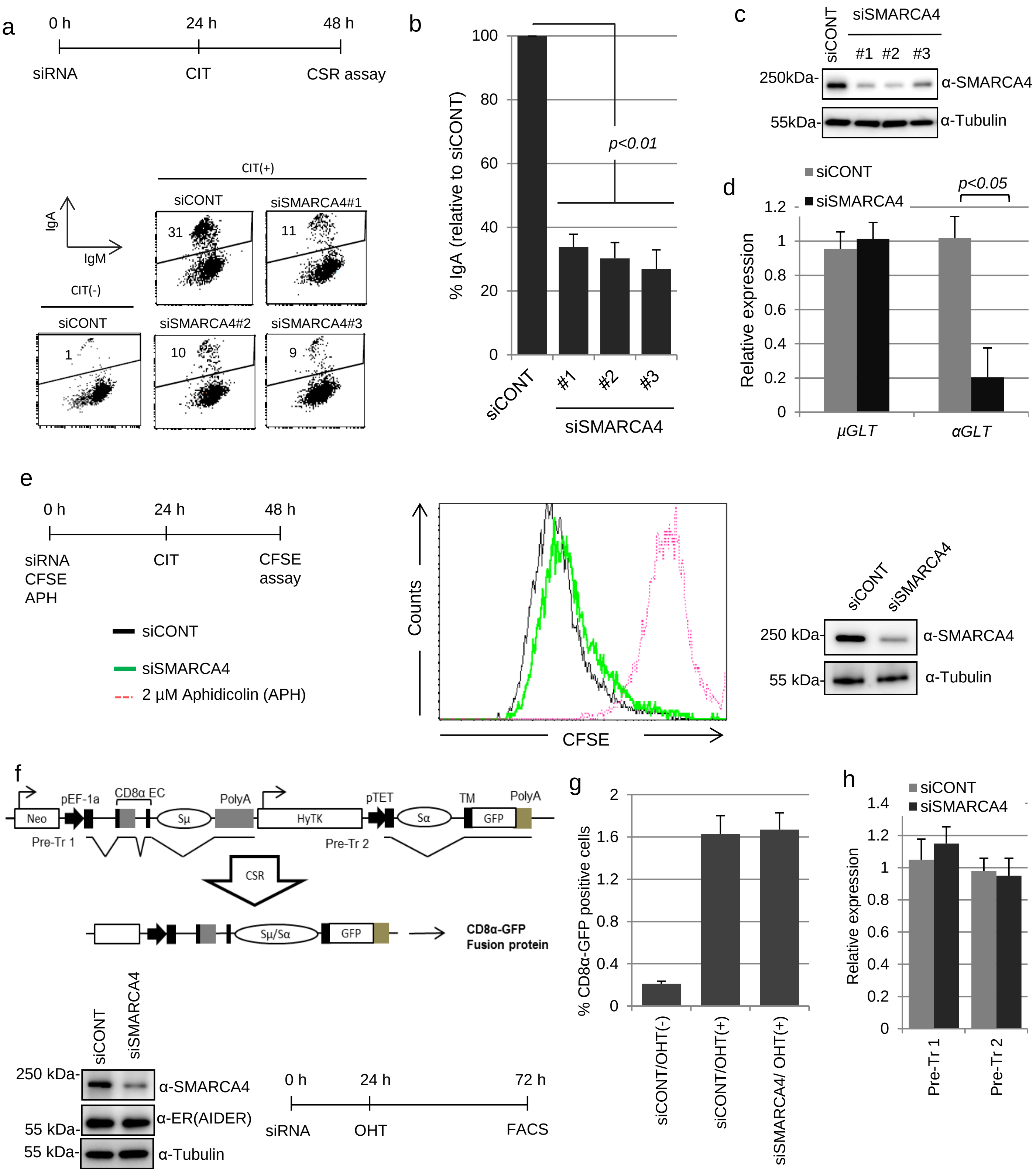

Supplementary Figure 2. SMARCA4 is required for efficient class switch recombination in B cells. (a) *Top:* Scheme of the IgA-switching assay in CH12F3-2A cells. After electroporation of siRNAs, cells were cultured for 24 h, and then stimulated by CIT, and cultured for another 24 h before FACS analysis. *Bottom:* FACS profile of the percentage of cells undergoing IgA switching following transfection of the indicated siRNAs into CH12F3-2A cells cultured with (+) or without (-) CIT stimulation for 24 h. The number in the FACS plot represents the percentage of the cells expressing IgA on their surface. (b) Summary of the FACS data derived from three independent experiments. Data is represented as % IgA switching relative to the IgA switching in cells transfected with control siRNA (siCONT) with similar GC content. The data represents the mean of the three independent experiments with standard deviations.

(c) Confirmation of SMARCA4 KD by siRNA in the CH12F3-2A cells. Tubulin is used as loading control. (d) Quantitative RT-PCR analysis of μGLT and αGLT expression from the indicated samples. The data were normalized to *Hprt*, and represents the mean of the two independent experiments with standard deviations. (e) The effect of SMARCA4 depletion on cell proliferation. *Left*: Scheme of the cell proliferation assay in CH12F3-2A. CH12F3-2A cells were transfected with siSMARCA4 or control siRNA (siCONT), and immediately labeled with CFSE (5 μM) for 15 min at 37°C. CIT was added 24 hours later and FACS analysis was done at 24 hour post-stimulation. Aphidicolin (2μg/ml), a well-known inhibitor of cell-cycle progression, was also utilized as a positive control. *Middle:* Histograms show CFSE dye dilution derived from the indicated samples. *Right:* Confirmation of siRNA mediated KD of SMARCA4. Tubulin is used as loading control. (f-h) Effect of SMARCA4 KD on the CSR in NIH 3T3 cells expressing artificial switch substrate substrate SCI (μ, α) and AIDER. (f) Structure of the artificial switch substrate substrate (*Top*), scheme of the CSR assay in NIH 3T3 cells (*Bottom-left*), and confirmation of SMARCA4 KD by siRNA (*Bottom-right*). (g) Percentages of CD8α-GFP positive cells derived from the FACS analysis of three independent experiments. (h) Quantitative RT-PCR analysis of expression of artificial switch transcripts (Pre-Tr1 and Pre-Tr2) from the indicated samples. Sequences of the primers used for transcript analysis is shown in Supplementary Table 8. The position of molecular weight markers is shown on the left of each western blot image. Statistical significance as evaluated by Student's *t*-test is shown. CIT, CD40L-IL4-TGFβ; CSR, class switch recombination; APH, Aphidicolin; CFSE, Carboxyfluorescein diacetate succinimidyl ester; AIDER, AID fused with the hormone-binding domain of the estrogen receptor (ER); GLT, germ line transcripts; OHT, 4-hydroxytamoxifen.

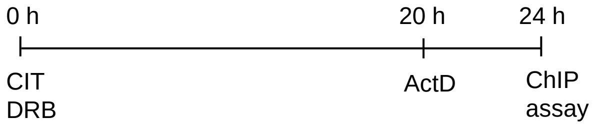

Mock=No antibody control
None=DMSO
DRB= 25μM of 5,6-Dichloro-1-β-D-ribofuranosylbenzimidazole for 24 h
ActD= 2μM of Actinomycin D for 4 h

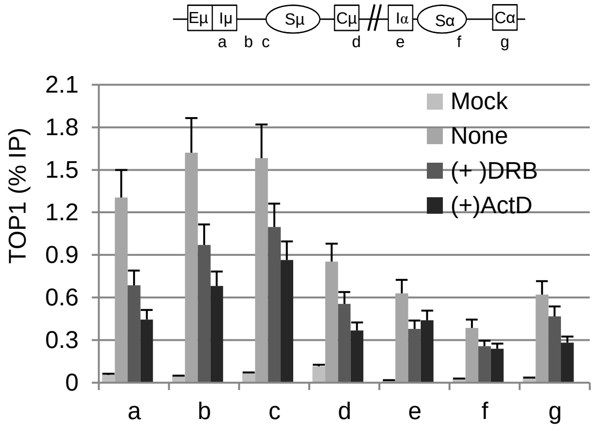

Supplementary Figure 3. Effect of transcription inhibitors (ActD or DRB) on TOP1 binding to chromatin, related to Fig. 2. CH12F3-2A cells undergoing CIT stimulation were treated with either 25 μM of DRB for 24 h or 2 μM of ActD for 4 h. Following treatment with these transcription inhibitors, cells were further treated with 10 μM of camptothecin for 30 min before lysis and ChIP analysis. ChIP data were normalized as in Fig. 3h. The mock data show background values from control IP with no antibody.

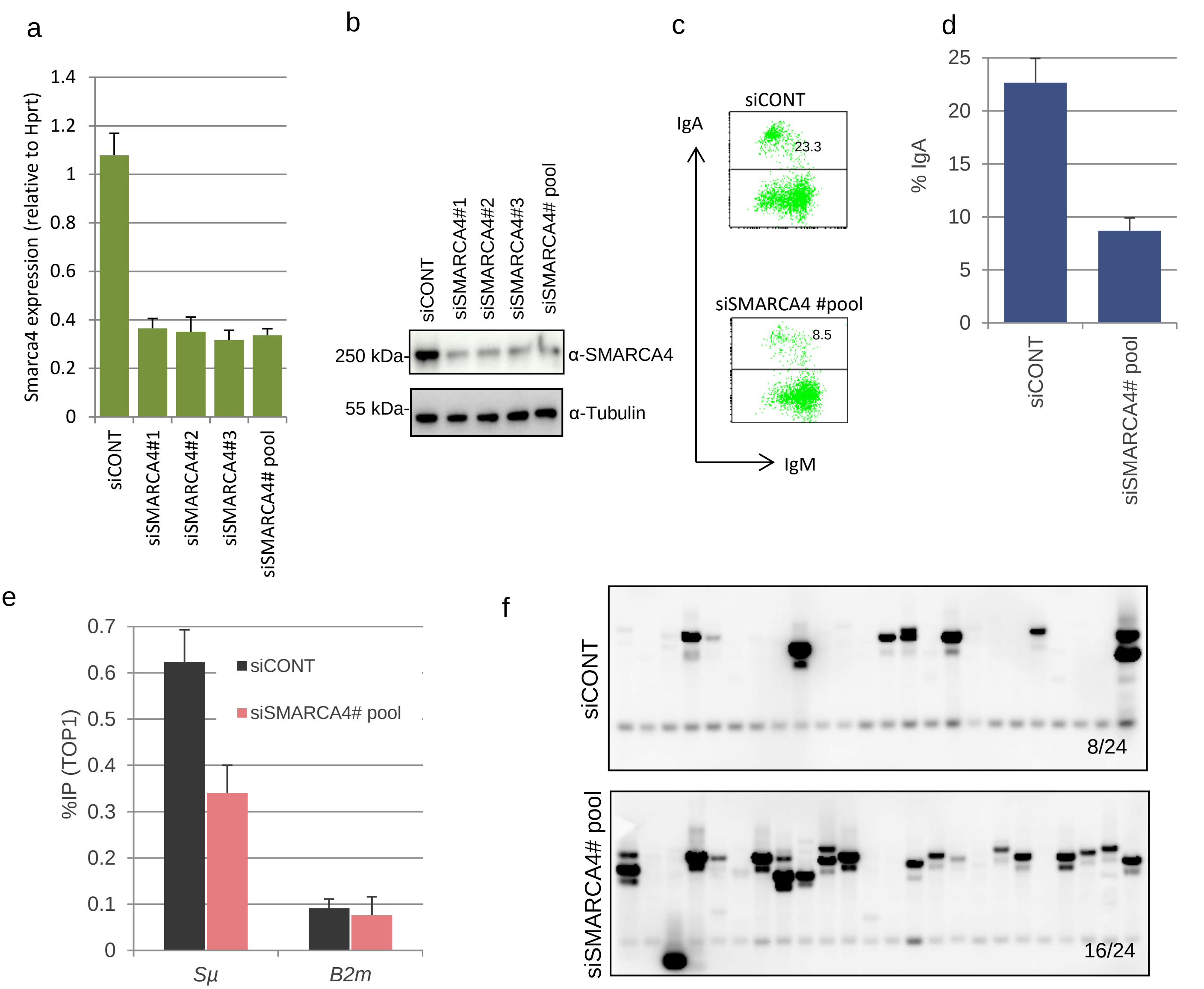

Supplementary Figure 4: Analysis of CSR, TOP1-recruitment, and *Igh/c-Myc* translocations upon SMARCA4 KD by a pool of three SMARCA4 siRNAs. Confirmation of SMARCA4 KD in the CH12F3-2A cells by individual or pool of three SMARCA4 siRNAs using quantitative RT-PCR (a) and Western blotting (b). The quantitative RT-PCR data were normalized to *Hprt*, and represents the mean of the two independent experiments with standard deviations. Tubulin is used as a loading control for Western blotting analysis. (c) FACS profile of the percentage of CH12F3-2A cells undergoing IgA switching following transfection of pool of three SMARCA4 siRNAs. After electroporation of siRNAs, cells were cultured for 24 h, and then stimulated by CIT, and cultured for another 24 h before FACS analysis. The number in the FACS plot represents the percentage of the cells expressing IgA on their surface. (d) Summary of the FACS data derived from two independent experiments. Data is represented as percentage of the cells expressing IgA on their surface. (e) Analysis of TOP1-ChIP in CH12F3-2A cells upon SMARCA4 KD by a pool of three SMARCA4 siRNAs. TOP1-ChIP signal in Sμ region is represented as the fraction of immunoprecipitated DNA (%IP) normalized to the total amount of DNA used for immunoprecipitation. The data represents the mean of the two independent experiments with standard deviations. (f) Analysis of *Igh/c-Myc* chromosomal translocations upon SMARCA4 KD by a pool of three SMARCA4 siRNAs. After electroporation of siRNAs, cells were cultured for 24 h, and then stimulated by CIT for another 48 h before southern blot analysis. The numbers at bottom-right corners indicate the number of the translocations detected in total number of PCR reactions. *B2m*, Beta-2-microglobulin.

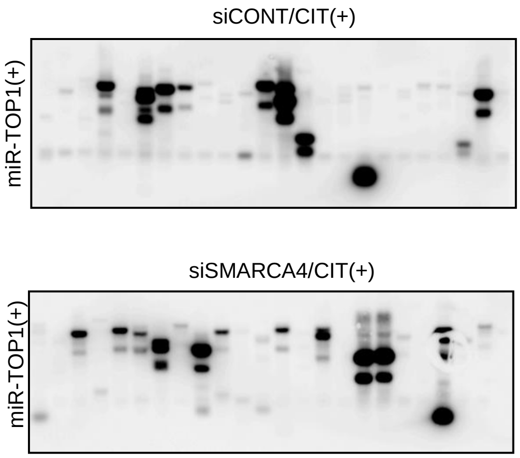

Supplementary Figure 5. Failure to detect augmentation in chromosomal translocations upon SMARCA4 KD in TOP1 depleted cells is not due to saturation of the assay, related to Fig. 4. The PCR-amplified fragments from genomic DNAs isolated from CH12F3-2A cells expressing Tet-inducible microRNA targeting *Top1* mRNA (miR-TOP1) following SMARCA4 siRNA transfection and tetracycline treatment (50 nM) were subjected to Southern blot analysis with *Myc*-specific probe. CIT, CD40L-IL4-TGFβ.

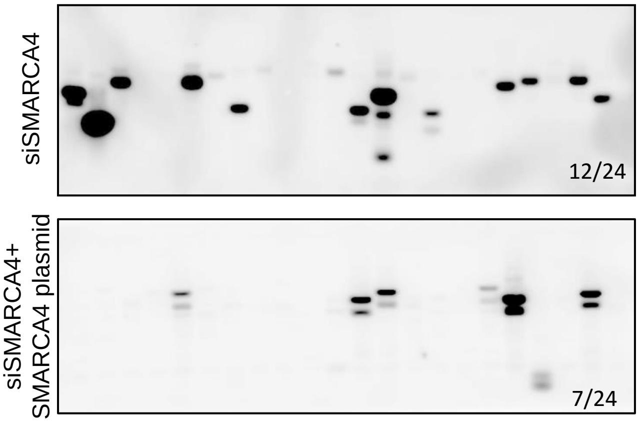

Supplementary Figure 6: Rescue of SMARCA4 KD induced enhancement in *Igh/c-Myc* translocations by WT SMARCA4. Analysis of *Igh/c-Myc* chromosomal translocations upon complementation of SMARCA4 KD by the transfection of siRNA-resistant GFP-tagged human SMARCA4. After co-transfection of SMARCA4 siRNA and siRNA-resistant GFP-tagged human SMARCA4 plasmid, cells were cultured for 24 h, and then stimulated by CIT for another 48 h before southern blot analysis with *Myc*-specific probe. The numbers at bottom-right corners indicate the number of the translocations detected in total number of PCR reactions. . CIT, CD40L-IL4-TGFβ.

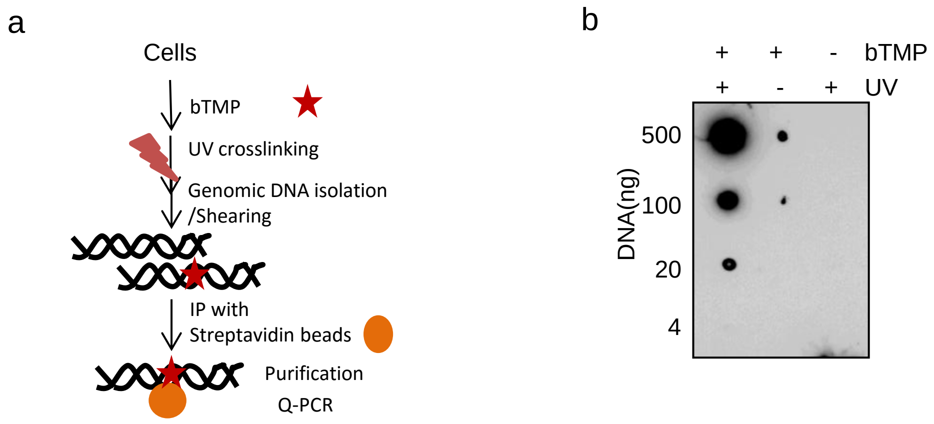

Supplementary Figure 7. Supporting information for the analysis of negative superhelicity, related to Fig. 6. (a) Schematic summary of the bTMP (biotin-trimethylpsoralen) ChIP assay. (b) Confirmation of the UV-dependent bTMP cross-linking of genomic DNA. The Indicated amount of the genomic DNA prepared from cells with indicated treatment were analyzed with dot-blot using HRP-conjugated Streptavidin as probe.

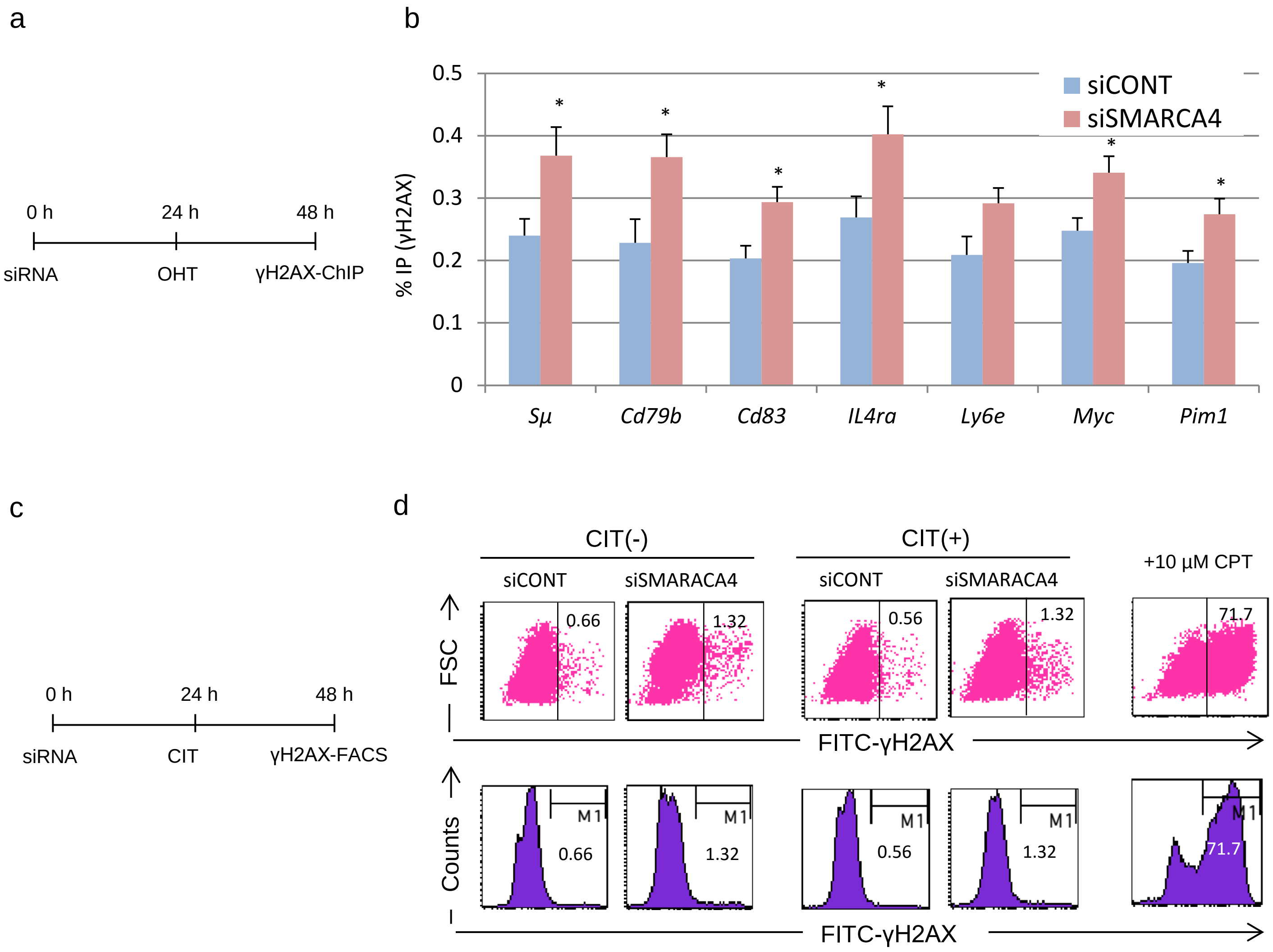

Supplementary Figure 8. Analysis of double strand break formation in SMARCA4 depleted CH12F3-2A cells. (a) Scheme of the γH2AX-ChIP assay in CH12F3-2A cells expressing the estrogen-binding domain (ER)-AID (AIDER) fusion protein. After electroporation of siRNAs, cells were cultured for 24 h, and then treated with OHT (1μ) for another 24 h before ChIP analysis. (b) Analysis of γH2AX-ChIP at *Igh* (Sμ) as well as non-*Igh* AID targets. The γH2AX-ChIP signal is shown as the fraction of immunoprecipitated DNA (%IP) normalized to the total amount of DNA used for immunoprecipitation. The data represents the mean of three independent experiments with standard deviations. *Asterisks* (*) denote statistically significant differences with $p \leq 0.05$, as determined by Student's *t*-test. (c) Scheme of the γH2AX-FACS staining in CH12F3-2A cells. After electroporation of siRNAs, cells were cultured for 24 h, and then stimulated by CIT for another 24 h before FACS staining. (d) FACS analysis of CH12F3-2A cells upon SMARCA4 KD in the presence (+) or absence (-) of CIT. As a positive control, cells were treated with 10 μM of camptothecin (CPT) for 3 h. The number in each dot plot or histogram shows the percentage of cells with high level of γH2AX. CIT, CD40L-IL4-TGFβ; CSR, class switch recombination; OHT, 4-hydroxytamoxifen.

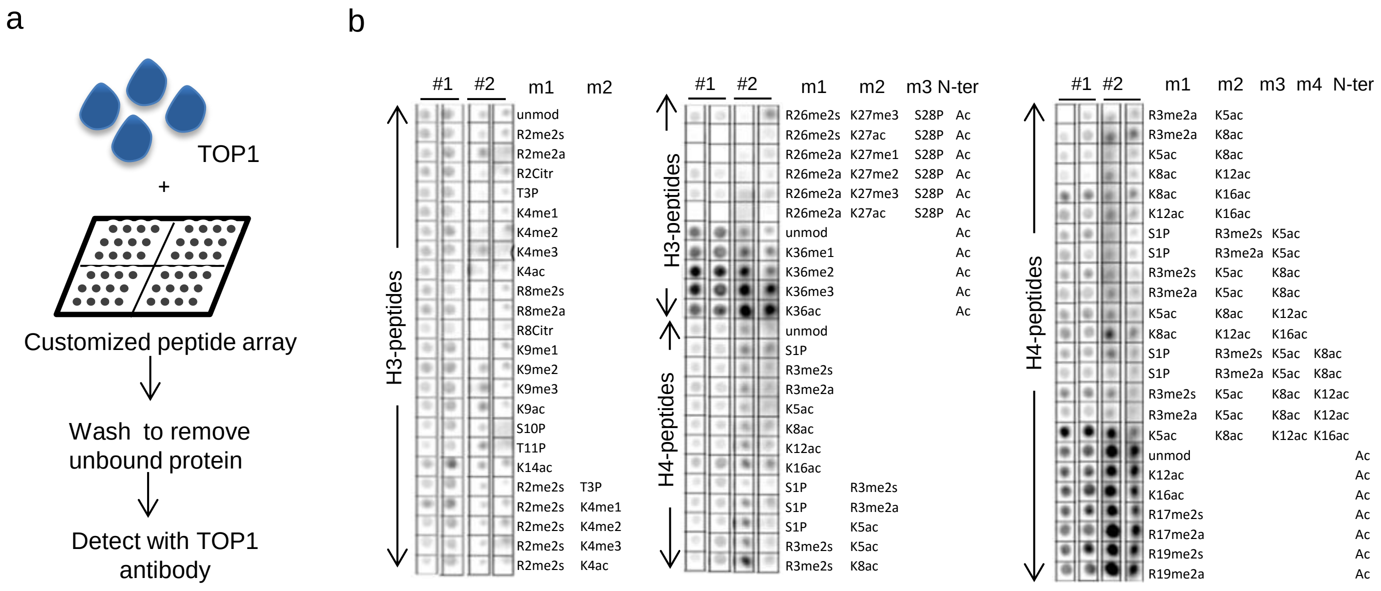

Supplementary Figure 9. Analysis of the direct binding of TOP1 to histone PTMs using custom-made peptide arrays and recombinant TOP1, related to Fig. 9. (a) Schematic summary of the peptide binding assay using custom-made peptide arrays (Active Motif). (b) Results of the peptide binding assay. Numbers 1 and 2 (#1, #2) represent data from two independent peptide arrays, whereas m1, m2, and m3 represent the different PTMs present on each peptide. N-ter shows the modification at the N-terminus of the peptide. PTMs, post-translational modifications.

Fig. 2b

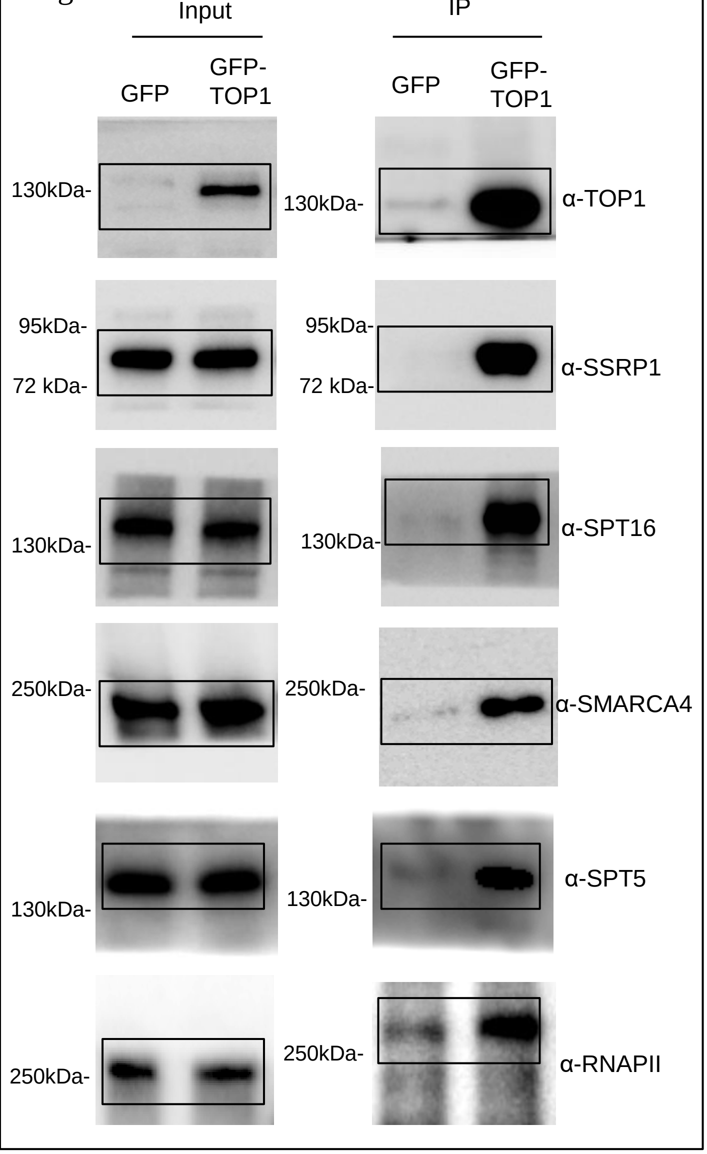

Fig. 2c

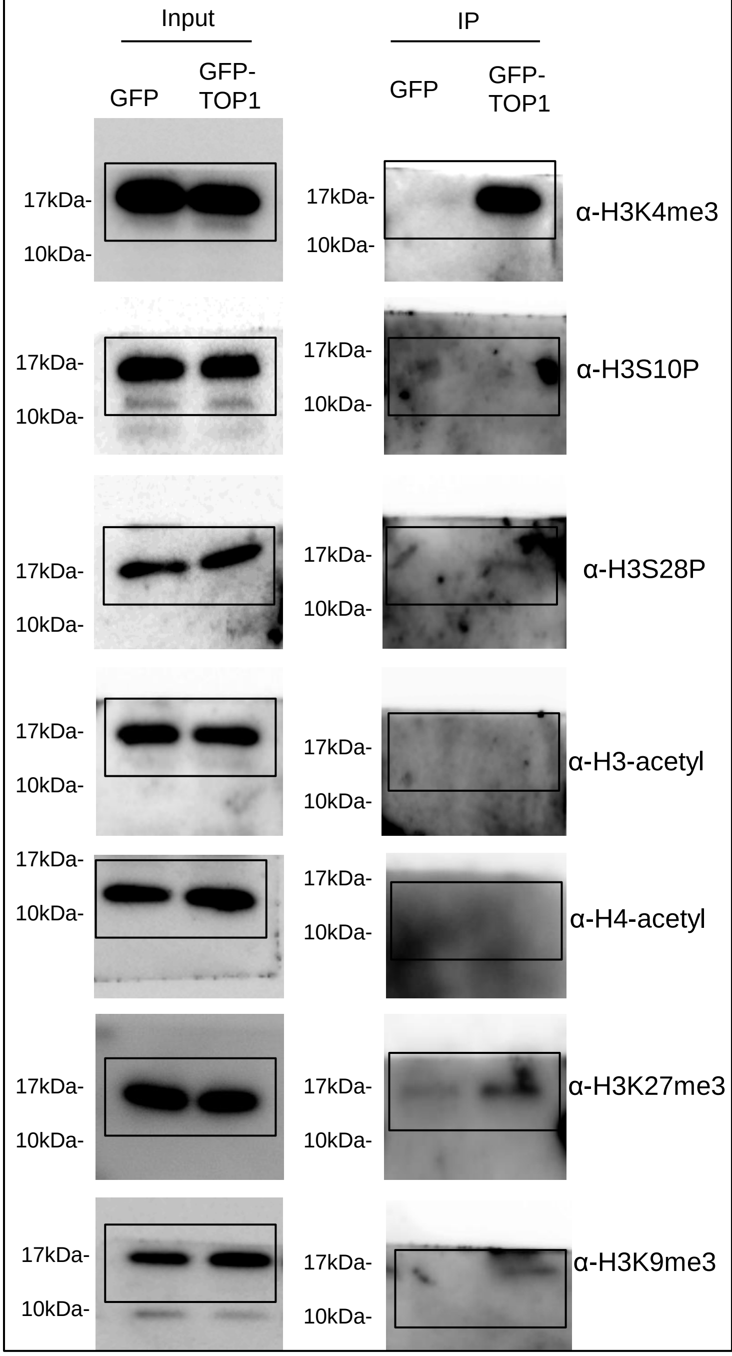

Fig. 3b

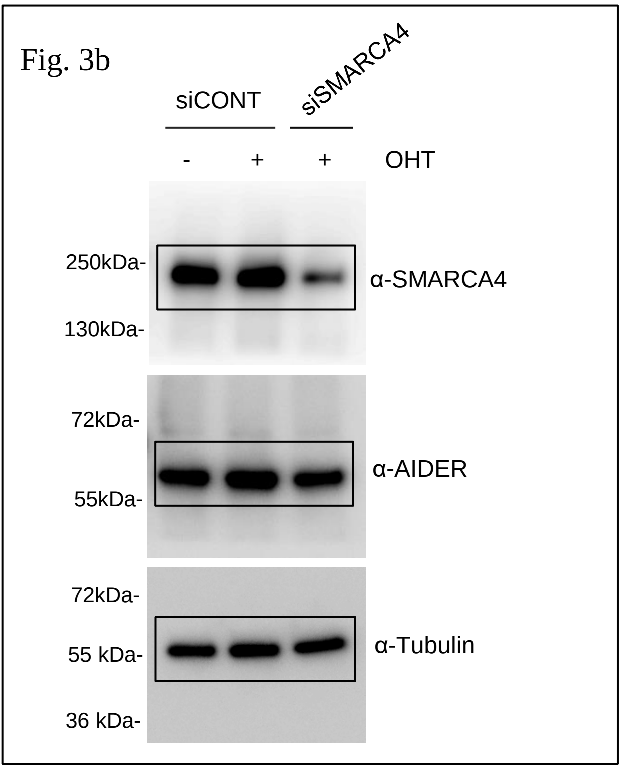

Fig. 3f

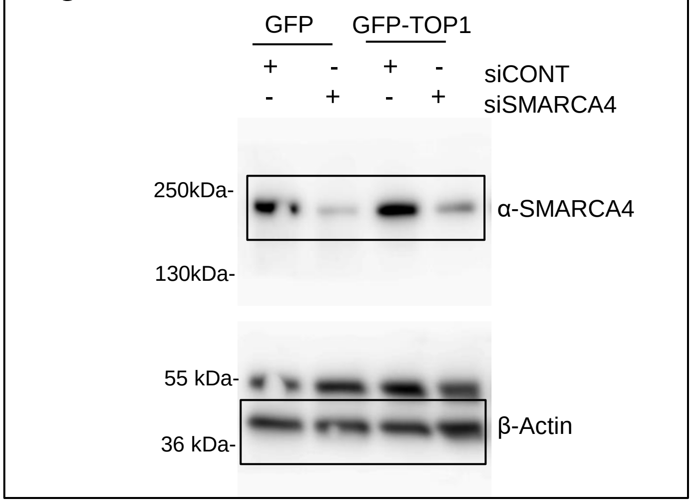

Supplementary Figure 10. Larger images of the most important western blots shown in the manuscript.

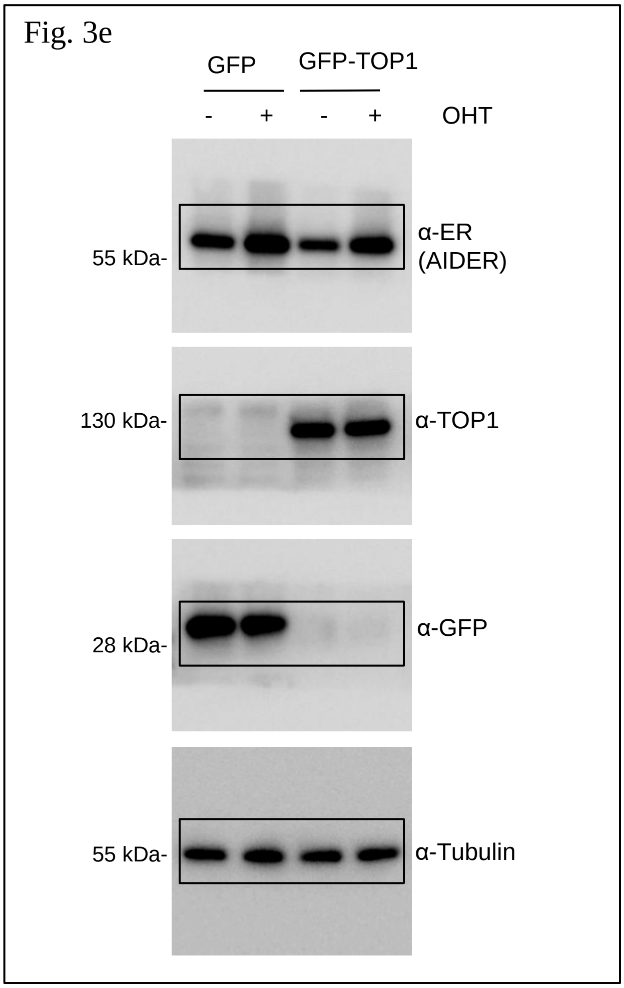

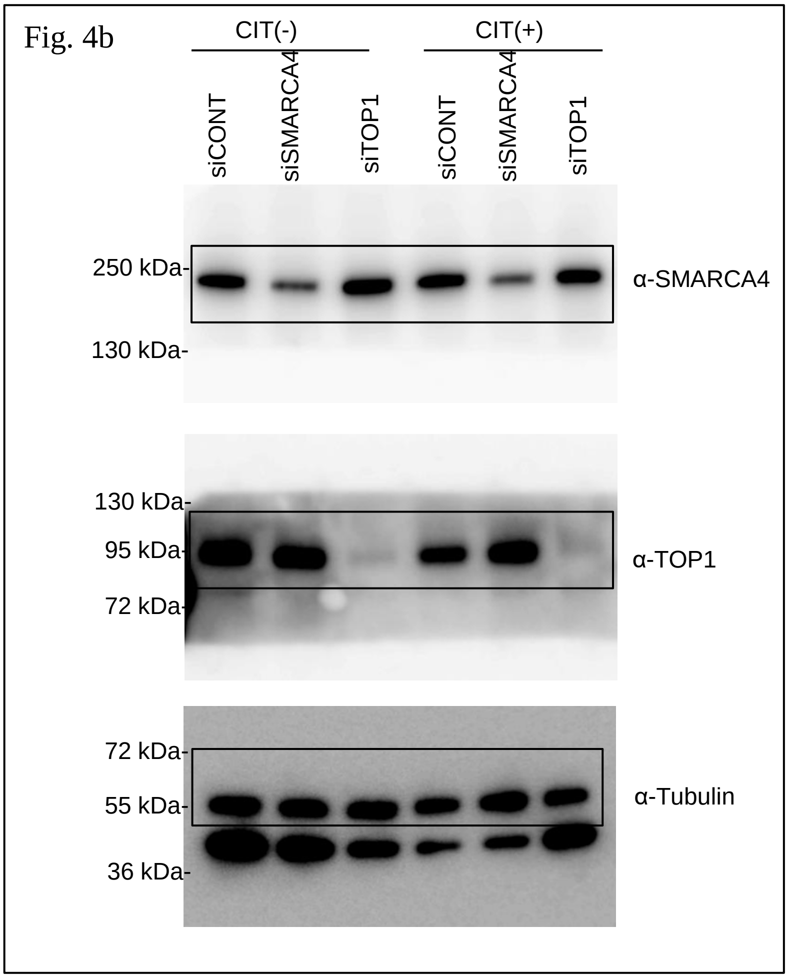

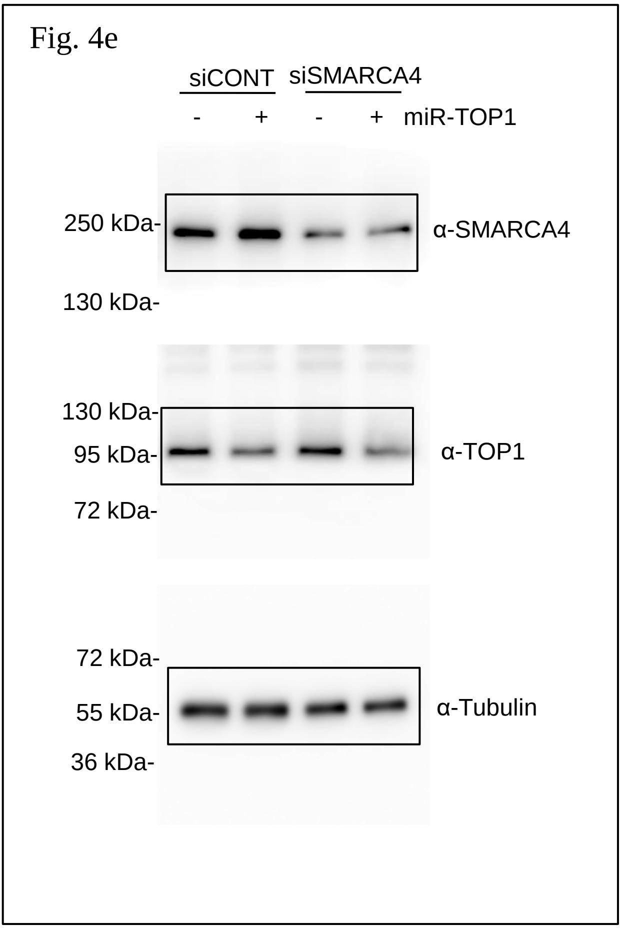

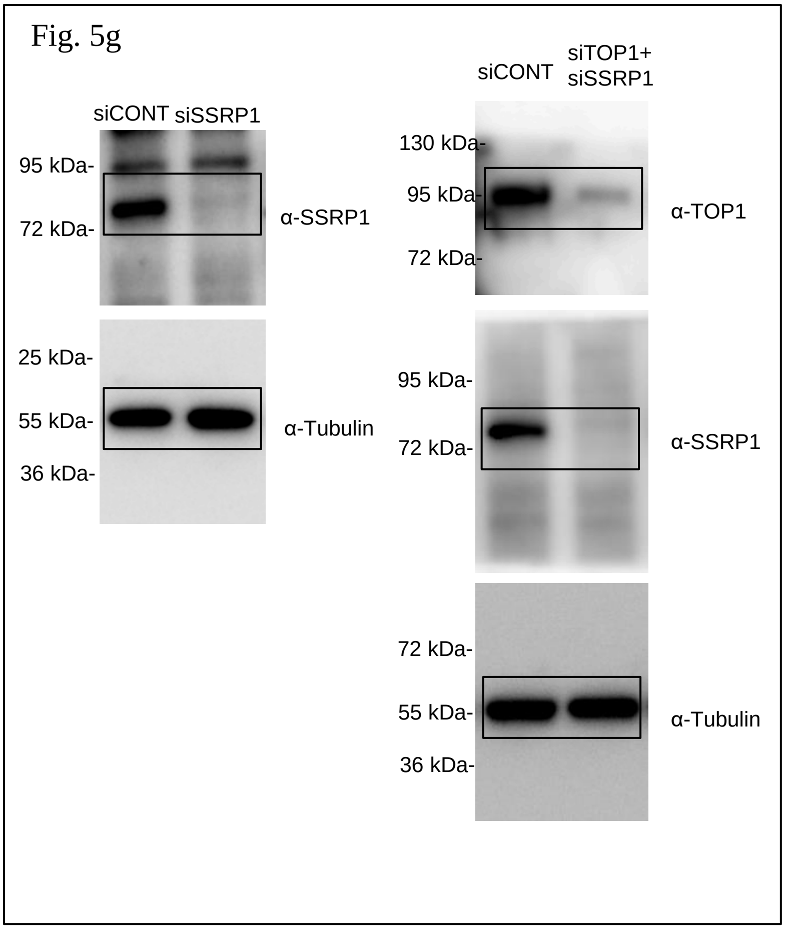

Supplementary Figure 10. Larger images of the most important western blots shown in the manuscript. *Continued..*

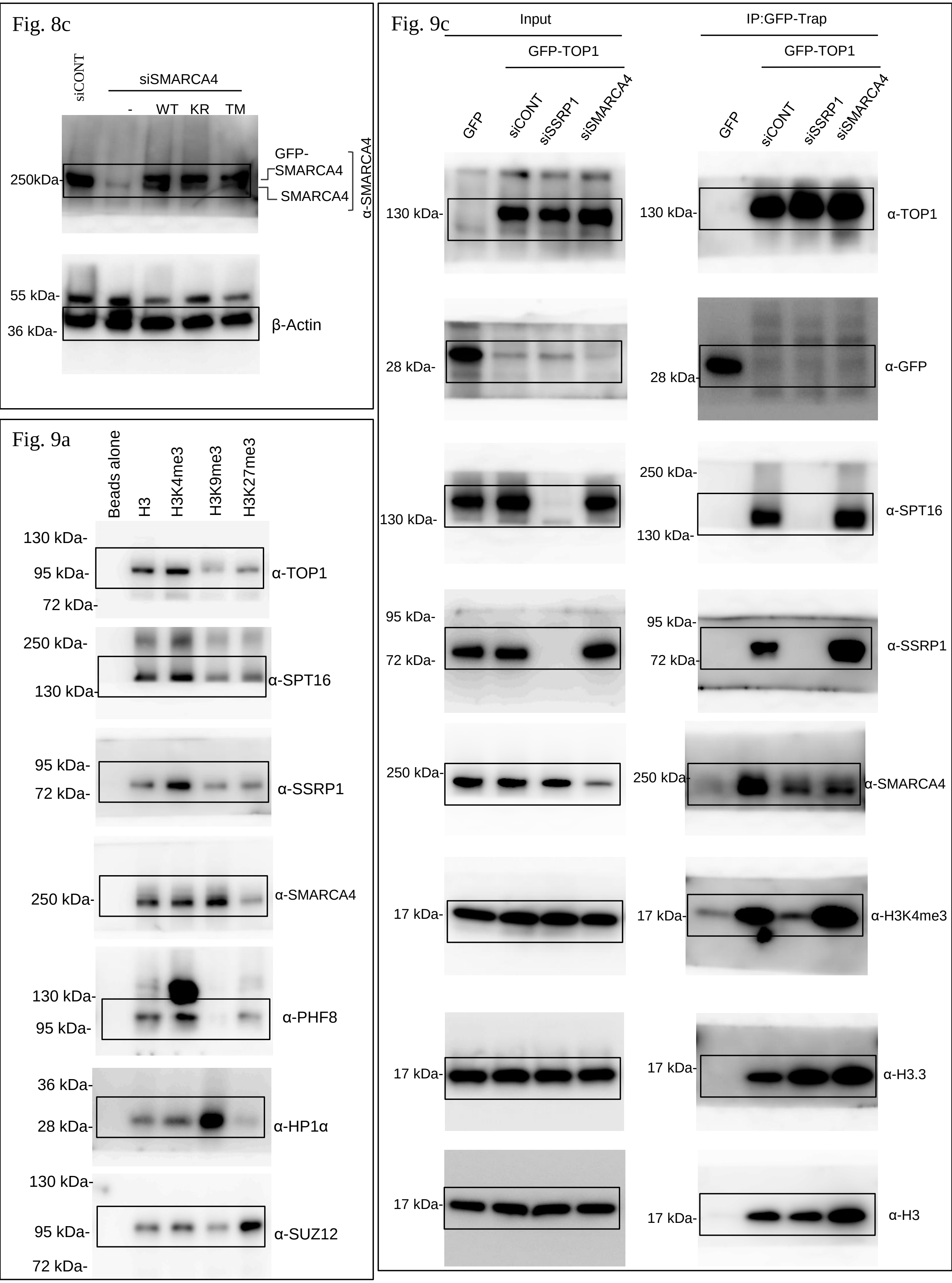

Supplementary Figure 10. Larger images of the most important western blots shown in the manuscript. *Continued..*

Supplementary Table 1. List of proteins with significant changes obtained in DSP-IP experiments. Data are averages of six measurements.

Protein Symbol	UniProt Accession No.	Protein Name	Mass	MASCOT Score	# of Petides used for Quantitation	Ratio (Average)	CV
Top1	TOP1_MOUSE	DNA topoisomerase 1 (bait)	90,819	10,983	126	58712.3	1.6
Top2a	TOP2A_MOUSE	DNA topoisomerase 2-alpha	172,683	2,935	28	612.4	2.3
Ddx21	DDX21_MOUSE	Nucleolar RNA helicase 2	93,494	2,357	28	27.0	0.3
Ncl	NUCL_MOUSE	Nucleolin	76,677	2,336	22	18.2	0.7
Ssrp1	SSRP1_MOUSE	FACT complex subunit SSRP1	80,811	1,609	18	268.7	1.0
Top2b	TOP2B_MOUSE	DNA topoisomerase 2-beta	181,796	1,131	13	52.2	1.6
Mybbp1a	MBB1A_MOUSE	Myb-binding protein 1A	151,942	1,462	13	22.1	0.6
Supt16h	SP16H_MOUSE	FACT complex subunit SPT16	119,750	1,569	11	103.7	1.2
Tcof1	TCOF_MOUSE	Treacle protein	134,921	789	11	54.4	0.9
Parp1	PARP1_MOUSE	Poly [ADP-ribose] polymerase 1	113,029	1,084	11	28.9	0.4
Trim28	TIF1B_MOUSE	Transcription intermediary factor 1-beta	88,791	1,305	10	18.2	0.7
Hmgb1	HMGB1_MOUSE	High mobility group protein B1	24,879	758	9	19.4	0.2
Sfpq	SFPQ_MOUSE	Splicing factor, proline- and glutamine-rich	75,395	864	9	11.0	0.4
Nop58	NOP58_MOUSE	Nucleolar protein 58	60,305	1,218	8	65.2	1.1
Prpf8	PRP8_MOUSE	Pre-mRNA-processing-splicing factor 8	273,443	1,070	8	22.0	0.4
Hirip3	HIRP3_MOUSE	HIRA-interacting protein 3	65,176	493	7	485.9	1.7
Dhx9	DHX9_MOUSE	ATP-dependent RNA helicase A	149,381	629	7	100.1	1.0
Chd4	CHD4_MOUSE	Chromodomain-helicase-DNA-binding protein 4	217,614	827	7	33.6	1.1
Dnmt1	DNMT1_MOUSE	DNA (cytosine-5)-methyltransferase 1	183,074	1,094	7	18.4	0.5
Matr3	MATR3_MOUSE	Matrin-3	94,572	887	7	18.2	0.5
Nono	NONO_MOUSE	Non-POU domain-containing octamer-binding protein	54,507	700	7	12.0	0.1
Thrap3	TR150_MOUSE	Thyroid hormone receptor-associated protein 3	108,114	544	6	85.9	0.6
Ikzf1	IKZF1_MOUSE	DNA-binding protein Ikaros	57,300	554	6	69.0	1.6
Smc3	SMC3_MOUSE	Structural maintenance of chromosomes protein 3	141,468	812	6	62.9	1.6
Prpf19	PRP19_MOUSE	Pre-mRNA-processing factor 19	55,205	852	6	27.8	1.4
Rbbp7	RBBP7_MOUSE	Histone-binding protein RBBP7	47,761	415	6	17.6	0.9
Runx1	RUNX1_MOUSE	Runt-related transcription factor 1	48,580	321	5	4472.8	2.2
Uhrf1	UHRF1_MOUSE	E3 ubiquitin-protein ligase UHRF1	88,248	486	5	61.6	1.9
Wdr43	WDR43_MOUSE	WD repeat-containing protein 43	75,334	453	5	45.2	0.4
Dkc1	DKC1_MOUSE	H/ACA ribonucleoprotein complex subunit 4	57,366	591	5	37.7	0.5
Rbmxl1	RMXL1_MOUSE	RNA binding motif protein, X-linked-like-1	42,137	357	5	25.3	0.7
Rbbp4	RBBP4_MOUSE	Histone-binding protein RBBP4	47,627	334	5	12.0	0.2
Sf3b1	SF3B1_MOUSE	Splicing factor 3B subunit 1	145,725	578	4	398.7	2.2
Smtn	SMTN_MOUSE	Smoothelin	100,228	320	4	186.7	1.6
Nat10	NAT10_MOUSE	N-acetyltransferase 10	115,346	401	4	178.2	1.1
Pds5a	PDS5A_MOUSE	Sister chromatid cohesion protein PDS5 homolog A	150,232	363	4	148.8	1.7
Smc1a	SMC1A_MOUSE	Structural maintenance of chromosomes protein 1A	143,147	553	4	80.5	1.6
Ubtf	UBF1_MOUSE	Nucleolar transcription factor 1	89,454	607	4	58.0	0.6
Rad21	RAD21_MOUSE	Double-strand-break repair protein rad21 homolog	72,038	312	4	52.5	1.3
Snrpd2	SMD2_MOUSE	Small nuclear ribonucleoprotein Sm D2	13,519	293	4	48.5	0.6
Actl6a	ACL6A_MOUSE	Actin-like protein 6A	47,418	368	4	48.3	0.9
Csnk2a1	CSK21_MOUSE	Casein kinase II subunit alpha	45,105	334	4	38.7	0.9
Rbm14	RBM14_MOUSE	RNA-binding protein 14	69,406	325	4	25.3	0.8
Mcm3	MCM3_MOUSE	DNA replication licensing factor MCM3	91,490	524	4	24.7	1.2
Fbl	FBRL_MOUSE	rRNA 2'-O-methyltransferase fibrillarin	34,286	498	4	14.6	0.2
Elavl1	ELAV1_MOUSE	ELAV-like protein 1	36,147	801	4	14.4	0.6
U2surp	SR140_MOUSE	U2 snRNP-associated SURP motif-containing protein	118,188	187	3	278.8	1.4
Smarcc1	SMRC1_MOUSE	SWI/SNF complex subunit SMARCC1	122,814	379	3	119.0	2.2
Smarca5	SMCA5_MOUSE	SWI/SNF-related matrix-associated regulator of chromatin su	121,551	393	3	99.4	1.2
Chd1	CHD1_MOUSE	Chromodomain-helicase-DNA-binding protein 1	196,264	305	3	41.9	0.4
Hdgfrp2	HDGR2_MOUSE	Hepatoma-derived growth factor-related protein 2	74,247	299	3	34.1	0.9
Mcm4	MCM4_MOUSE	DNA replication licensing factor MCM4	96,676	264	3	25.8	0.5
Arid1a	ARI1A_MOUSE	AT-rich interactive domain-containing protein 1A	241,940	321	3	20.7	0.4
Keap1	KEAP1_MOUSE	Kelch-like ECH-associated protein 1	69,508	42	2	6733233.4	2.2
Sub1	TCP4_MOUSE	Activated RNA polymerase II transcriptional coactivator p15	14,419	248	2	170.6	0.8
Zfp62	ZFP62_MOUSE	Zinc finger protein 62	104,744	153	2	108.7	0.5

Fanci	FANCI_MOUSE	Fanconi anemia group I protein homolog	149,231	137	2	84.6	0.9
Supt6h	SPT6H_MOUSE	Transcription elongation factor SPT6	198,962	221	2	79.3	0.8
Supt5h	SPT5H_MOUSE	Transcription elongation factor SPT5	120,590	280	2	78.8	1.8
Adnp	ADNP_MOUSE	Activity-dependent neuroprotector homeobox protein	92,006	257	2	76.1	1.2
Snrnp40	SNR40_MOUSE	U5 small nuclear ribonucleoprotein 40 kDa protein	39,251	646	2	66.4	1.2
Safb2	SAFB2_MOUSE	Scaffold attachment factor B2	111,771	167	2	62.3	0.9
Plrg1	PLRG1_MOUSE	Pleiotropic regulator 1	56,903	316	2	35.4	0.3
Csnk2b	CSK2B_MOUSE	Casein kinase II subunit beta	24,927	222	2	32.2	0.6
Srsf10	SRS10_MOUSE	Serine/arginine-rich splicing factor 10	31,282	259	2	29.4	0.6
Psip1	PSIP1_MOUSE	PC4 and SFRS1-interacting protein	59,661	208	2	28.8	0.3
Xrcc1	XRCC1_MOUSE	DNA repair protein XRCC1	68,929	231	2	23.8	0.5
Utp6	UTP6_MOUSE	U3 small nucleolar RNA-associated protein 6 homolog	70,385	172	2	18.1	0.6
Mcm5	MCM5_MOUSE	DNA replication licensing factor MCM5	82,291	351	2	15.9	0.5
Ssb	LA_MOUSE	Lupus La protein homolog	47,727	495	2	15.6	0.3
Pak1ip1	PK1IP_MOUSE	p21-activated protein kinase-interacting protein 1	42,090	288	2	15.4	0.4
Stag2	STAG2_MOUSE	Cohesin subunit SA-2	141,191	245	2	14.8	0.3
Eed	EED_MOUSE	Polycomb protein EED	50,166	103	2	13.5	0.2
No66	NO66_MOUSE	Lysine-specific demethylase NO66	67,515	101	2	10.0	0.1
Smarca4	SMCA4_MOUSE	Transcription activator BRG1	181,314	89	1	15.7	0.3
Pax5	PAX5_MOUSE	Paired box protein Pax-5	42,206	131	1	6548.6	1.7
Polr2b	RPB2_MOUSE	DNA-directed RNA polymerase II subunit RPB2	133,825	152	1	1589.0	2.0
Baz1a	BAZ1A_MOUSE	Bromodomain adjacent to zinc finger domain protein 1A	178,349	167	1	191.0	1.5
Champ1	CHAP1_MOUSE	Chromosome alignment-maintaining phosphoprotein 1	87,506	64	1	167.2	1.6
Prmt1	ANM1_MOUSE	Protein arginine N-methyltransferase 1	42,408	309	1	84.7	0.8
Brd4	BRD4_MOUSE	Bromodomain-containing protein 4	155,826	151	1	58.7	1.7
Sin3a	SIN3A_MOUSE	Paired amphipathic helix protein Sin3a	144,998	104	1	55.7	0.8
Baz1b	BAZ1B_MOUSE	Tyrosine-protein kinase BAZ1B	170,544	139	1	36.6	0.5
U2af1	U2AF1_MOUSE	Splicing factor U2AF 35 kDa subunit	27,798	136	1	27.2	0.7
Smc6	SMC6_MOUSE	Structural maintenance of chromosomes protein 6	127,118	92	1	26.3	0.7
Fiz1	FIZ1_MOUSE	Flt3-interacting zinc finger protein 1	52,652	112	1	25.6	0.4
Srrt	SRRT_MOUSE	Serrate RNA effector molecule homolog	100,391	368	1	24.1	0.5
Phf6	PHF6_MOUSE	PHD finger protein 6	41,113	287	1	21.9	0.5
Wdr18	WDR18_MOUSE	WD repeat-containing protein 18	47,182	169	1	21.8	0.6

Supplementary Table 2. List of proteins with significant changes obtained in native-IP experiments.

Protein Symbol	UniProt Accession No.	Protein Name	Mass	P388/CPT45-GFP-TOP1		P388/CPT45-GFP		# unique peptides	Ratio
				#1	#2	#1	#2		
Top1	TOP1_MOUSE	DNA topoisomerase 1(bait)	90,819	28,098	32,109	99	794	128	67.4
Ssrp1	SSRP1_MOUSE	FACT complex subunit SSRP1	80,810	6,709	8,153	N.D.	31	42	247.7
Supt6h	SPT6H_MOUSE	Transcription elongation factor SPT6	198,962	1,383	3,537	N.D.	N.D.	33	84.8
Sub1	TCP4_MOUSE	Activated RNA polymerase II transcriptional coactivator p15	14,418	2,140	2,286	N.D.	N.D.	12	76.3
Smarca4	SMCA4_MOUSE	Transcription activator BRG1	181,313	149	253	N.D.	N.D.	3	6.9
Lig3	DNLI3_MOUSE	DNA ligase 3	113,000	2,173	1,799	N.D.	N.D.	24	68.5
Polb	DPOLB_MOUSE	DNA polymerase beta	38,264	1,994	1,513	N.D.	N.D.	25	60.5
Xrcc1	XRCC1_MOUSE	DNA repair protein XRCC1	68,929	2,663	2,370	62	N.D.	14	55.3
Polr2a	RPB1_MOUSE	DNA-directed RNA polymerase II subunit RPB1	217,039	2,311	2,875	N.D.	71	29	51.9
Pdcd11	RRP5_MOUSE	Protein RRP5 homolog	207,649	1,410	1,531	N.D.	N.D.	15	50.7
Lbr	LBR_MOUSE	Lamin-B receptor	71,395	972	1,687	N.D.	N.D.	6	45.8
Supt16h	SP16H_MOUSE	FACT complex subunit SPT16	119,749	8,659	9,767	68	350	52	44.1
Prpf8	PRP8_MOUSE	Pre-mRNA-processing-splicing factor 8	273,443	2,556	4,722	69	149	58	33.4
Pole	DPOE1_MOUSE	DNA polymerase epsilon catalytic subunit A	261,932	469	1,376	N.D.	N.D.	19	31.8
U2af2	U2AF2_MOUSE	Splicing factor U2AF 65 kDa subunit	53,483	1,419	1,454	30	62	15	31.2
Pelp1	PELP1_MOUSE	Proline-, glutamic acid- and leucine-rich protein 1	117,995	787	989	N.D.	N.D.	14	30.6
U2af1	U2AF1_MOUSE	Splicing factor U2AF 35 kDa subunit	27,797	866	900	N.D.	N.D.	8	30.4
Safb2	SAFB2_MOUSE	Scaffold attachment factor B2	111,770	723	962	N.D.	N.D.	12	29.1
Snrnp200	U520_MOUSE	U5 small nuclear ribonucleoprotein 200 kDa helicase	244,392	3,782	5,376	115	217	54	27.6
Srsf1	SRSF1_MOUSE	Serine/arginine-rich splicing factor 1	27,728	1,155	1,420	67	N.D.	14	26.8
Srsf3	SRSF3_MOUSE	Serine/arginine-rich splicing factor 3	19,318	1,243	1,351	72	N.D.	11	25.7
Tdp1	TYDP1_MOUSE	Tyrosyl-DNA phosphodiesterase 1	68,647	661	710	N.D.	N.D.	8	23.6
Srsf10	SRS10_MOUSE	Serine/arginine-rich splicing factor 10	31,282	560	787	N.D.	N.D.	5	23.2
Eif4a3	IF4A3_MOUSE	Eukaryotic initiation factor 4A-III	46,810	427	903	N.D.	N.D.	11	22.9
Snrnp40	SNR40_MOUSE	U5 small nuclear ribonucleoprotein 40 kDa protein	39,251	687	639	N.D.	N.D.	8	22.9
Mcm6	MCM6_MOUSE	DNA replication licensing factor MCM6	92,809	467	681	N.D.	N.D.	10	19.8
Dkc1	DKC1_MOUSE	H/ACA ribonucleoprotein complex subunit 4	57,365	470	609	N.D.	N.D.	6	18.6
Parp1	PARP1_MOUSE	Poly [ADP-ribose] polymerase 1	113,028	8,262	9,152	407	552	55	18.2
Nop58	NOP58_MOUSE	Nucleolar protein 58	60,305	528	517	N.D.	N.D.	6	18.0
Aqr	AQR_MOUSE	Intron-binding protein aquarius	170,185	443	511	N.D.	N.D.	5	16.4
Wdr43	WDR43_MOUSE	WD repeat-containing protein 43	75,334	477	429	N.D.	N.D.	6	15.6
Chtop	CHTOP_MOUSE	Chromatin target of PRMT1 protein	26,568	499	353	N.D.	N.D.	6	14.7
Prpf19	PRP19_MOUSE	Pre-mRNA-processing factor 19	55,204	852	840	88	N.D.	10	14.5
Plrg1	PLRG1_MOUSE	Pleiotropic regulator 1	56,902	350	467	N.D.	N.D.	6	14.1
Thrap3	TR150_MOUSE	Thyroid hormone receptor-associated protein 3	108,114	437	361	N.D.	N.D.	7	13.8
Bclaf1	BCLF1_MOUSE	Bcl-2-associated transcription factor 1	105,939	256	537	N.D.	N.D.	8	13.7
Tex10	TEX10_MOUSE	Testis-expressed sequence 10 protein	105,143	334	443	N.D.	N.D.	5	13.4
Srrm1	SRRM1_MOUSE	Serine/arginine repetitive matrix protein 1	106,798	296	470	N.D.	N.D.	9	13.2
Srsf9	SRSF9_MOUSE	Serine/arginine-rich splicing factor 9	25,645	318	424	N.D.	N.D.	7	12.8
Smu1	SMU1_MOUSE	WD40 repeat-containing protein SMU1	57,507	336	397	N.D.	N.D.	5	12.6
Srsf5	SRSF5_MOUSE	Serine/arginine-rich splicing factor 5	30,873	288	433	N.D.	N.D.	2	12.4
Nop56	NOP56_MOUSE	Nucleolar protein 56	64,424	342	370	N.D.	N.D.	4	12.3
Senp3	SENP3_MOUSE	Sentrin-specific protease 3	64,362	394	305	N.D.	N.D.	4	12.1
Srrm2	SRRM2_MOUSE	Serine/arginine repetitive matrix protein 2	294,666	351	337	N.D.	N.D.	6	11.9
Cpsf1	CPSF1_MOUSE	Cleavage and polyadenylation specificity factor subunit 1	160,716	446	237	N.D.	N.D.	5	11.8
Mcm7	MCM7_MOUSE	DNA replication licensing factor MCM7	81,160	217	443	N.D.	N.D.	6	11.4
Wdr18	WDR18_MOUSE	WD repeat-containing protein 18	47,181	336	309	N.D.	N.D.	3	11.1
Cbx5	CBX5_MOUSE	Chromobox protein homolog 5	22,172	311	325	N.D.	N.D.	5	11.0
Hdgfrp2	HDGR2_MOUSE	Hepatoma-derived growth factor-related protein 2	74,246	370	258	N.D.	N.D.	5	10.8
Thoc2	THOC2_MOUSE	THO complex subunit 2	182,658	281	338	N.D.	N.D.	3	10.7
Gins1	PSF1_MOUSE	DNA replication complex GINS protein PSF1	22,879	245	330	N.D.	N.D.	4	9.9
Gins3	PSF3_MOUSE	DNA replication complex GINS protein PSF3	24,561	304	263	N.D.	N.D.	4	9.8
H2afy	H2AY_MOUSE	Core histone macro-H2A.1	39,710	2,159	1,941	177	245	27	9.7
Wdr61	WDR61_MOUSE	WD repeat-containing protein 61	33,752	318	234	N.D.	N.D.	3	9.5
Tra2b	TRA2B_MOUSE	Transformer-2 protein homolog beta	33,646	1,170	1,284	159	102	6	9.4
Nipbl	NIPBL_MOUSE	Nipped-B-like protein	315,253	213	354	N.D.	33	5	9.1
Rbm8a	RBM8A_MOUSE	RNA-binding protein 8A	19,876	196	325	N.D.	N.D.	4	9.0
Tra2a	TRA2A_MOUSE	Transformer-2 protein homolog alpha	32,297	220	276	N.D.	N.D.	1	8.6
Rbbp7	RBBP7_MOUSE	Histone-binding protein RBBP7	47,760	189	298	N.D.	N.D.	4	8.4
Chd4	CHD4_MOUSE	Chromodomain-helicase-DNA-binding protein 4	217,614	995	2,758	196	289	29	7.7
Pnkp	PNKP_MOUSE	Bifunctional polynucleotide phosphatase/kinase	57,188	202	209	N.D.	N.D.	3	7.1
Cdc73	CDC73_MOUSE	Parafibromin	60,539	165	240	N.D.	N.D.	4	7.0
Btbd1	BTBD1_MOUSE	BTB/POZ domain-containing protein 1	53,199	141	258	N.D*.	N.D.	2	6.9
Rbm14	RBM14_MOUSE	RNA-binding protein 14	69,406	256	328	56	N.D.	3	6.9

Samples were first separated with SDS-PAGE and peptides obtained by in-gel digestion were analyzed twice with LC/MS/MS. N.D.: not detected. For ratio calculation score of 29 was used for proteins not detected, as the score cutoff for MASCOT search was set to 30.

Supplementary Table 3. Mutation analysis of the V region in BL2-AID$^{-/-}$AIDER cells.

Condition	Mutated/ Total Clones	Total Mutations	Substitutions	Ins/ Dels	Total bases	Mutations/ base (x10^{-4})
siCONT/OHT(-)	3/89	3	3	0	36312	0.83
siCONT/OHT(+)	29/233	35	32	3	95064	3.37
siSMARCA4/OHT(+)	43/223	53	51	2	90984	5.61

Del, deletion; Ins, insertion; OHT, 4-hydroxytamoxifen

Supplementary Table 4. Base substitution patterns in the V region of BL2-AID$^{-/-}$AIDER cells .

siCONT OHT(+)		To: G	C	A	T	Total	%
From	G		2	11	1	14	91
	C	2			13	15	
	A	2				2	9
	T	1				1	

siSMARCA4 OHT(+)		To: G	C	A	T	Total	%
From	G		4	20	2	26	88
	C	4		2	13	19	
	A				1	1	12
	T			5		5	

Supplementary Table 5. Mutation analysis of the Sμ region in P388/CPT45 cells.

Cells	Condition	Mutated/ Total clones	Total Mutations	Substitutions	Ins/Del	Total bases	Mutations/ base ($x10^{-4}$)
P388/CPT45-GFP	siCONT/OHT(-)	0/29	0	0	0	17690	0.00
	siCONT/OHT(+)	15/120	20	15	5	73200	2.05
	siSMARCA4/OHT(+)	13/126	17	15	2	76860	1.95
P388/CPT45-GFP-TOP1	siCONT/OHT(-)	0/27	0	0	0	16470	0.00
	siCONT/OHT(+)	7/126	9	6	3	76860	0.78
	siSMARCA4/OHT(+)	18/125	21	18	3	76250	2.36

Del, deletion; Ins, insertion; OHT, 4-hydroxytamoxifen

Supplementary Table 6. Base substitution patterns in the Sμ region of P388/CPT45 cells.

P388/CPT45-GFP cell

siCONT/OHT(+)

From \ To	G	C	A	T	Total	%
G			6		6	93
C	1			7	8	
A	1				1	7
T						

siSMARCA4/OHT(+)

From \ To	G	C	A	T	Total	%
G			10		10	93
C			1	3	4	
A						7
T		1			1	

P388/CPT45-GFP-TOP1 cells

siCONT/OHT(+)

From \ To	G	C	A	T	Total	%
G			1		1	83
C	1		1	2	4	
A	1				1	17
T						

siSMARCA4/OHT(+)

From \ To	G	C	A	T	Total	%
G		1	6		7	89
C	1		2	6	9	
A	1				1	11
T			1		1	

Supplementary Table 7. Summary of the effect of SMARCA4, FACT, or TOP1 KD on AID-induced genetic alterations.

	TOP1 KD	SMARCA4 KD	FACT KD
TOP1 level at chromatin	↓	↓	unchanged
Non-B DNA structures	↑	↑	unchanged
Hypermutagenesis	↑	↑	↓
Chromosomal translocations	↑	↑	↓
TOP1-H3K4me3 complex	ND	unchanged	↓

The upwards or downwards pointing arrows show increments or decrements of the measured parameters. ND, not determined.

Supplementary Table 8. List of primers used in the present study.

CH12F3-2 cells	
**ChIP analysis**	
a-F	AAGGGCTTCTAAGCCAGTCC
a-R	CACAACCATACATTCCCAGGT
b-F	AAAGAGACATTTGTGTGTCTTTGAGTACCG
b-R	ATTGGTTAACAGGCAACATTTTTCTTTTAC
c-F	GTATCAAAGGACAGTGCTTAGATCCAAGGT
c-R	TTTCTCAATTCTGTACAGCTGTGGCCTTCC
d-F	CAGCACCATTTCCTTCACCTGGAACTACCA
d-R	GGCTAGGTACTTGCCCCCTGTCCTCAGTGT
e-F	GTGATTCAGGGAGCAAGAGC
e-R	TCTAGCCTGGGAGTCTCCTG
f-F	TGAAAAGACTTTGGATGAAATGTGAACCAA
f-R	GATACTAGGTTGCATGGCTCCATTCACACA
g-F	AGTGCCCAGAAGGAGAATCCGTGAAATGTT
g-R	GACCCCCTAACGTTCTTTACCAGAGCAATT
Actb-F	CAGCTTCTTTGCAGCTCCTT
Actb-R	CTAGCCACGAGAGAGCGAAG
B2m-F	CTGGCTGGCTCTCATTTCAG
B2m-R	GGTCAGTGAGACAAGCACCA
Gapdh-F	ATCCTGTAGGCCAGGTGATG
Gapdh-R	AGGCTCAAGGGCTTTTAAGG
Eef1a1-F	AGTCGCCTTGGACGTTCTT
Eef1a1-R	GGGAATGCTCGCAGCTAAT
Tcrd-F	GGAGTGTTTCACCACACTTGCC
Tcrd-R	TTGCCAAGACCATTGCCATC
Ly6e-F	TCCTTCCTGCACGTTTCTGGA
Ly6e-R	CTGCTCTAGTTTACGAGGGTGT
IL4ra-F	TGTGGGCGAGAGAACAACTTCG
IL4ra-R	GCTGAGGCATGGATCTGTTAGG
Cd79b-F	CACTCTTCCAGAGCAAGGCAAC
Cd79b-R	GGGAAGGGGTTGCTCCTGAATC
Cd83-F	GCCTCCAGCTCCTGTTTCTAG
Cd83-R	GGCTGCAGGCTACAAGAGAGA
Pim1-F	CATCACCCCCAGGATCTAGCC
Pim1-R	GAAGCCATGAGTAGGGACCGC
Myc-F	CGCGATCAGCTCTCCTGAAAAG
Myc-R	GAACACAGGGAAAGACCACCAG
**Transcript analysis**	
μGLT-F	CTCTGGCCCTGCTTATTGTTG
μGLT-R	AATGGTGCTGGGCAGGAAGT
αGLT-F	CCAGGCATGGTTGAGATAGAGATAG
αGLT-R	GAGCTGGTGGGAGTGTCAGTG

Hprt-F	CTCGAAGTGTTGGATACAGG
Hprt-R	TGGCCTATAGGCTCATAGTG
**IgH/c-Myc chromosomal translocations**	
Sμ5’1a (first)	ACTATGCTATGGACTACTGGGGTCAAG
c-Myc5’1a (first)	GTGAAAACCGACTGTGGCCCTGGAA
Sμ5′1b (second)	CCTCAGTCACCGTCTCCTCAGGTA
c-Myc5′1b (second)	GTGGAGGTGTATGGGGTGTAGAC
c-Myc probe	DIG-GGACTGCGCAGGGAGACCTACAGGGG
**LM-PCR**	
LMPCR.1	GCGGTGACCCGGGAGATCTGAATTC
LMPCR.2	GAATTCAGATC
5′Sμ-F	GCAGAAAATTTAGATAAAATGGATACCTCAGTGG
5′Sμ-probe	DIG-AGGGACCCAGGCTAAGAAGGCAAT
**BL2 cells**	
**V region mutation**	
V-seq-F	CTATAACCATGGTTCATGAAACACCTGTGGTTC
V-seq-R	TGCATGCATTCTAGAAAGGGTTGGGGCGGATGCACTCC
**P388/CPT45 cells**	
**S region mutation**	
Sμ-seq-F	GGAATTCGTAAGGAGGGACCCAGGCTAAG
Sμ-seq-R	GAATTCCAGTCCAGTGTAGGCAGTAGA
**NIH 3T3 cells**	
**Transcript analysis**	
Pre-Tr1-F	GGTTTGCCGCCAGAACACAG
Pre-Tr1-R	GATTTCTTGTCTCCCACGT
Pre-Tr2-F	CCACGCTGTTTTGACCTCCATGACA
Pre-Tr2-R	GTGGTTTGTCCAAACTCATCAATGT
**SMARCA4 plasmids**	
SMARCA4(WT)-Sal1-F	GGAATTCGTAAGGAGGGACCCAGGCTAAG
SMARCA4(WT)-EcoRV-R	GAATTCCAGTCCAGTGTAGGCAGTAGA
SMARCA4(K785R)-F	AGATGGGCCTGGGGAGGACCATCCAGACCAT
SMARCA4(K785R)-R	ATGGTCTGGATGGTCCTCCCCAGGCCCATCT
SMARCA4(T910M)-F	GCCGCCTGCTGCTGATGGGCACACCGCTGCA
SMARCA4(T910M)-R	TGCAGCGGTGTGCCCATCAGCAGCAGGCGGC

Supplementary Table 9. Information related to antibodies used in the present study.

Antibody	Company	Product No	Dilution/Amount
SMARCA4	Santa Cruz	sc-17796	700
SMARCA4	Millipore	07-478	1000
SSRP1(ChIP assay)	Biolegend	609702	3 μg
SSRP1	Abcam	ab26229	1000
SPT16	Santa Cruz	sc-377028	500
TOP1(ChIP assay)	Santa Cruz	sc-5342	3 μg
TOP1	LifeSpan Biosciences	LS-C39023	1000
TOP1	Abcam	ab109374	2000
γH2AX (ChIP assay)	Millipore	05-636	3 μg
H3K4me3	Millipore	07-473	2000
H3-acetyl	Active Motif	39139	2000
H4-acetyl	Active Motif	39243	2000
H3S28P	Millipore	07-145	2000
H3K9me3	Millipore	07-442	2000
H3.3	Millipore	09-838	3000
H3K27me3	Abcam	ab6002	2000
PHF8	Millipore	09-868	2000
GFP	Invitrogen	A11122	4000
HIRIP3	Abcam	ab100856	1000
H3	Abcam	ab1791	3000
BAZ1B	Abcam	ab50850	1000
CHD4	Abcam	ab72418	1000
XRCC1	Abcam	ab9147	1000
β-Actin	Sigma	A1978	4000
Tubulin	Calbiochem	CP06	3000
SUZ12	Cell Signaling	D39F6	1000
DNA LIGIII	Santa Cruz	sc-135883	700
SPT5	Santa Cruz	sc-28678	500
HP1α	Santa Cruz	sc-28735	3000
SRSF1	Santa Cruz	sc-33652	1000
ER	Santa Cruz	sc-787	1000
RNAPII	Santa Cruz	sc-9001	1000

Supplementary Table 10. List of siRNA oligonucleotides used in the present study.

Gene name	Stealth siRNA Official ID (Invitrogen)
Smarca4	MSS209195 (#1)
	MSS209196 (#2)
	MSS209197 (#3)
Top1	MSS212033
Ssrp1	MSS209558
Spt6	MSS209821
Chd4	MSS200895
Rbbp7	MSS281012
SMARCA4(human)	HSS185959
siControl	12935-300

For SMARCA4 KD in mouse cells oligo#3 was used in all of the experiments except CSR rescue assay in which oligo#1 was used.

Supplementary Table 11. List of biotinylated histone peptides used in peptide pull down assays.

Histone peptide	Company	Catalgue No
H3K4me3	ANASPEC	AS-64357-1
H3K9me3	ANASPEC	AS-64360-1
H3K27me3	ANASPEC	AS-64367-1
